# New approaches and technical considerations in detecting outlier measurements and trajectories in longitudinal children growth data

**DOI:** 10.1186/s12874-023-02045-w

**Published:** 2023-10-13

**Authors:** Paraskevi Massara, Arooj Asrar, Celine Bourdon, Moses Ngari, Charles D. G. Keown-Stoneman, Jonathon L. Maguire, Catherine S. Birken, James A. Berkley, Robert H. J. Bandsma, Elena M. Comelli

**Affiliations:** 1 Department of Nutritional Sciences, Faculty of Medicine, University of Toronto, Toronto, Canada; 2 Translational Medicine Program, Hospital for Sick Children, Toronto, Canada; 3 Kenya Medical Research Institute (KEMRI)/ Wellcome Trust Research Programme, Kilifi, Kenya; 4 Li KaShing Knowledge Institute, Unity Health Toronto, Toronto, Canada; 5 Dalla Lana School of Public Health, University of Toronto, Toronto, Canada; 6 Department of Pediatrics, Faculty of Medicine, University of Toronto, Toronto, Canada; 7 Child Health Evaluative Services, Hospital for Sick Children, Toronto, Canada; 8 Centre for Tropical Medicine and Global Health, Nuffield Department of Clinical Medicine, University of Oxford, Oxford, UK; 9 Joannah and Brian Lawson Center for Child Nutrition, University of Toronto, Toronto, Canada

**Keywords:** Growth outliers, Clustering, Growth measurements, Trajectories

## Abstract

**Background:**

Growth studies rely on longitudinal measurements, typically represented as trajectories. However, anthropometry is prone to errors that can generate outliers. While various methods are available for detecting outlier measurements, a gold standard has yet to be identified, and there is no established method for outlying trajectories. Thus, outlier types and their effects on growth pattern detection still need to be investigated. This work aimed to assess the performance of six methods at detecting different types of outliers, propose two novel methods for outlier trajectory detection and evaluate how outliers affect growth pattern detection.

**Methods:**

We included 393 healthy infants from The Applied Research Group for Kids (TARGet Kids!) cohort and 1651 children with severe malnutrition from the co-trimoxazole prophylaxis clinical trial. We injected outliers of three types and six intensities and applied four outlier detection methods for measurements (model-based and World Health Organization cut-offs-based) and two for trajectories. We also assessed growth pattern detection before and after outlier injection using time series clustering and latent class mixed models. Error type, intensity, and population affected method performance.

**Results:**

Model-based outlier detection methods performed best for measurements with precision between 5.72-99.89%, especially for low and moderate error intensities. The clustering-based outlier trajectory method had high precision of 14.93-99.12%. Combining methods improved the detection rate to 21.82% in outlier measurements. Finally, when comparing growth groups with and without outliers, the outliers were shown to alter group membership by 57.9 -79.04%.

**Conclusions:**

World Health Organization cut-off-based techniques were shown to perform well in few very particular cases (extreme errors of high intensity), while model-based techniques performed well, especially for moderate errors of low intensity. Clustering-based outlier trajectory detection performed exceptionally well across all types and intensities of errors, indicating a potential strategic change in how outliers in growth data are viewed. Finally, the importance of detecting outliers was shown, given its impact on children growth studies, as demonstrated by comparing results of growth group detection.

**Supplementary Information:**

The online version contains supplementary material available at 10.1186/s12874-023-02045-w.

## Introduction

Postnatal growth is a continuous and dynamic process that extends from birth until early adulthood [1–3]. Longitudinally growth monitoring aims to evaluate children’s nutritional and health status, with growth-monitoring programs being a critical part of pediatric health care and public health programs [4–8]. While utilization of longitudinal measurements is crucial, it entails data-cleaning challenges related to the temporality and the unique nature of child growth. First, outliers in growth data have natural relations with previous and subsequent measurements [9]. Moreover, the natural variations in body fat and lean mass proportion during physical development and various clinical conditions, such as edema, can affect measurements. These anomalies can create extreme outliers (potentially biologically implausible values or BIVs) or “milder” outliers that deviate from the main core of measurements while being potentially plausible [10, 11]. While BIVs can be detected using standard thresholds such as those provided by the World Health Organization (WHO) [12], “milder” outliers are more challenging because of their unclear definition and effects on the statistical analyses [13, 14].

Various methods exist for detecting outliers in growth measurements. The WHO growth standards cut-offs (i.e. +5/-5 for body mass index-for-age z-scores) detect BIVs in static measurements [12]. However, the growth standards aim to describe how children ‘should’ grow, not how they ‘do’ grow under non-optimal settings [15], and they do not account for the growth points before or after the potential outlier measurement. Other outlier detection methods consider the longitudinal nature of growth. Residuals post-model-fit and influential observations in a model assessment can be used for outlier detection. Other methods include the representation of trajectories within the context of a whole dataset for outlier visual assessment [16, 17], and future growth prediction approaches that detect outliers by comparing them against predicted values derived from children’s previously collected data [9, 18–21]. Limitations of these approaches include low sensitivity (i.e., the proportion of true outlier measurements correctly identified as outliers), specific requirements for a minimum number of measurements per-subject trajectory to be available and the focus on detecting outlier measurements instead of entire trajectories. Even though visual assessment [16, 17] can be used to detect entire outlier trajectories, this approach is impractical when analyzing larger epidemiological datasets. A more practical approach to detect outlier trajectories is crucial because trajectories are essential tools for growth monitoring.

Clustering-based techniques are an important category of outlier detection methods [22–27], under the hypothesis that extreme or irrelevant cases are further away from the main core of data and thus more isolated. However, the use of clustering for detecting outliers in the domain of human growth still needs to be explored. We previously [28] tested the performance of the clustering-based Multi-Model Outlier Measurement Detection method (MMOM) versus the modified method for biologically implausible values detection (mBIV), which is adapted for longitudinal measurements and is based on the WHO fixed cut-offs [29]. While both methods accounted for the longitudinal nature of growth measurements, MMOM performed better at identifying three different types of synthetic outliers [28]. This previous work focused only on two outlier measurement detection methods, one population, and one error intensity for the three types of injected outliers. Here we studied two child populations with different nutritional statuses (malnutrition vs normal or accelerating growth) to evaluate the applicability of outlier detection methods, not only on a measurement level but also on a trajectory level, focusing mainly on clustering-based techniques. Further, we assessed the effect of different outlier intensities on the performance of outlier detection methods and determined the impact of outliers on growth pattern detection.

## Methods

### Datasets

Two datasets, corresponding to two child populations, were studied. The first included 2,354 infants from The Applied Research Group for Kids (TARGet Kids!) cohort (www.clinicaltrials.gov, NCT01869530) [30]. TARGet Kids! is the largest ongoing primary healthcare-based network in Canada that recruits children from birth to 5 years from the Greater Toronto Area (Ontario, Canada). According to the provincial immunization and developmental screening schedule, children visit the pediatrician at ages 2, 4, 6, 9, 12, 18 and 24 months, with an additional post-partum screening visit scheduled within the first 30 days of life [30]. During these visits, weight and length are measured following established procedures [31]. Age- and sex-standardized weight-for-length values (zWFL) were generated using the WHO Child Growth Standards (2016) [12].

The second dataset included 1,955 children from the co-trimoxazole (CTX) prophylaxis trial (www.clinicaltrials.gov, NCT00934492) [32]. CTX was a randomized, double-blind, placebo-controlled trial that recruited children aged between 60 days and 59 months with severe malnutrition from four hospitals in Kenya. Anthropometry was conducted at enrolment, once per month for up to 6 months, and then twice a month from 6 to 12 months [32]. Age- and sex-standardized values for weight, and mid-upper arm circumference (MUAC) measures (zWA, zMUAC, respectively) were generated using the WHO Child Growth Standards (2016) [12]. Data included in both datasets were doubly entered, checked, and previously cleaned. In this perspective, detection accuracy was assessed only based on the artificially entered (injected synthetic) outliers and not on any previously existing outliers, as these were removed as part of the data cleaning process. This was done to create a controlled dataset and ensure certainty about the outlier detection.

### Experimental design

**Fig. 1:**
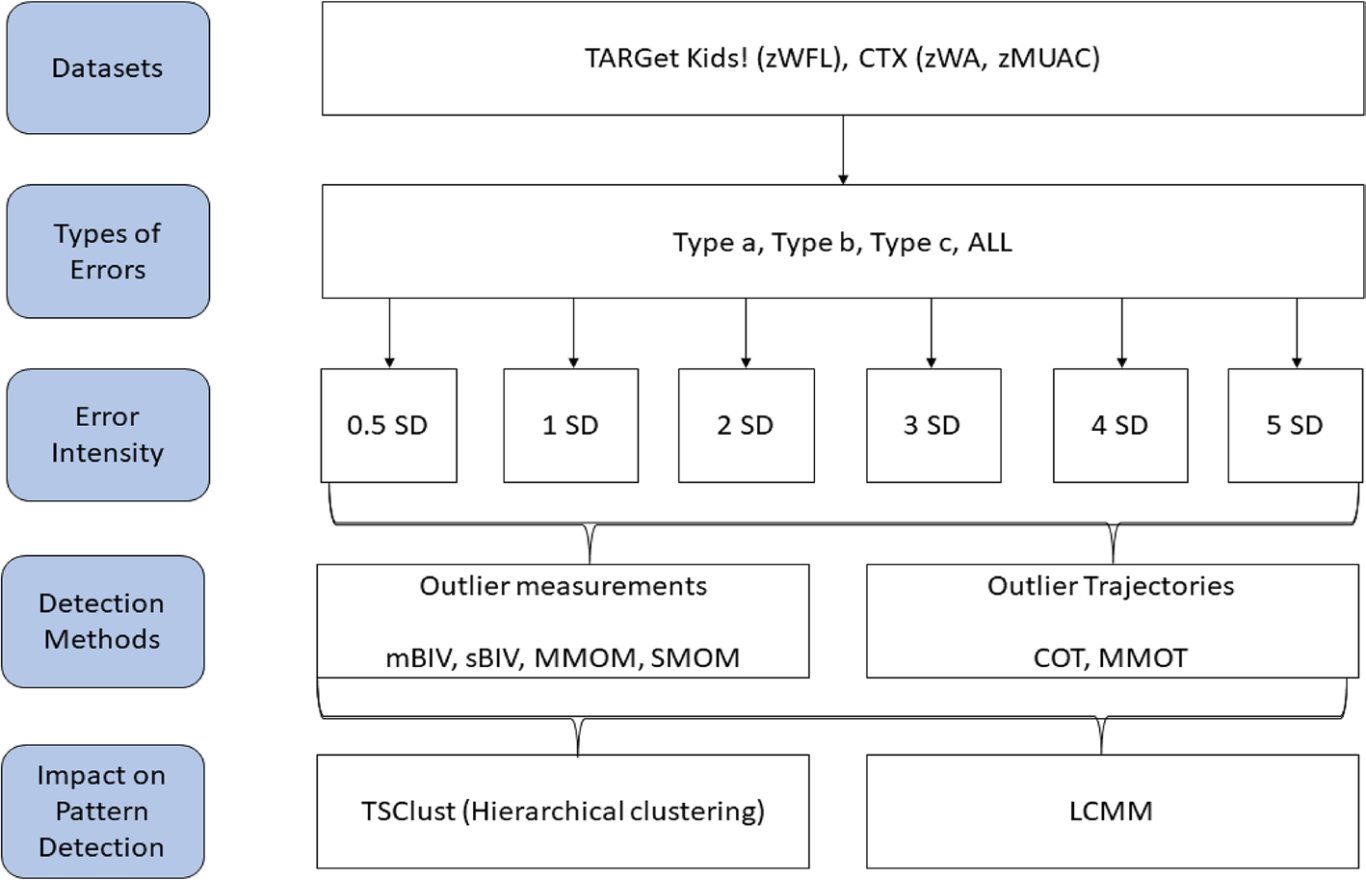
Study experimental design. The study involves 3 steps: a) the injection of synthetic outliers of different types and different intensities, b) the application of the outlier detection methods for outlier measurements and outlier trajectories, and c) the evaluation of the impact of outliers on growth pattern detection. Abbreviations: TARGet Kids!, the applied research group for kids; zWFL ; weight-for-length z-scores ; zWA, weight-for-age z-scores; CTX, the co-trimoxazole prophylaxis trial; zMUAC, mid-upper arm circumference-for-age z-scores; SD, standard deviation; mBIV, modified method for biologically implausible values detection; sBIV, static detection method for biologically implausible values based on fixed WHO cut-off values ; MMOM, multi-model outlier measurement detection method; SMOM, single-model outlier measurement detection method; COT, clustering-based outlier trajectory detection method; MMOT, multi-model outlier trajectory detection method

As shown in Fig. 1, we applied six outlier detection methods, four for single time-point outliers and two for trajectory outliers (Supplementary section 1), which were compared based on their ability to detect the respective kind of outliers for both child population and growth measures. For single time-point outliers, we used: 1) a static BIV detection method based on the fixed WHO cut-off values (sBIV) [12], 2) a modified BIV detection method for longitudinal measurements using the WHO cut-off values (mBIV), 3) a multi-model outlier measurement detection method based on clustering (MMOM) [12], and 4) a single-model outlier measurement detection method (SMOM). For trajectory outliers, we used: 1) a clustering outlier trajectory detection method based on hierarchical clustering (HC) (COT) and 2) a multi-model outlier trajectory detection method designed to consider sub-groups of growth trajectories (MMOT). Next, we generated three types of synthetic errors randomly in both datasets to create global (exceed the WHO standards) and contextual (within the context of an individual child) [33] outliers: moderate to extreme (Type a), extreme (Type b), local (Type c), and all types combined (ALL) [20]. This comparison was conducted for four different scenarios: (i) a dataset with type a errors only, (ii) a dataset with type b errors only, (iii) a dataset with type c errors only, and (iv) a dataset with types a, b and c. If a measurement was previously altered, another measurement was chosen at random. We also generated 6 different intensities, between 0.5 and 5 standard deviations (SDs), for each type: Type a was created by adding a positive (+) or negative (-) error of a standard normal distribution (μ=0, σ=1) to random measurements; Type b was created by adding a positive (+) error between 0.5 SD and 5 SD of a standard normal distribution (μ=0, σ=1) to the *absolute value* of random measurements (resulting in measurements greater than 3 or 4 z-scores); Type c was created by adding ± times the SD of an individual’s trajectory to random measurements of that specific individual (Supplementary Section 1). Outliers were injected in 5% of the dataset measurements for each type of error, resulting in 15% outliers for ALL types which were generated by adding up type of errors a, b, c (more details in Supplementary Section 1). The synthetic outliers were flagged and used as the “gold standard” for each of the six evaluation methods . The simulation experiments were conducted for each dataset for the different types of errors and different error intensities The outlier simulations were repeated 100 times.

### Outlier detection methods (Table 1)

**Table 1:** Key characteristics of the employed outlier detection methods

Method	**Key characteristics**			
	**Type of detection method**	**Types of outliers**	**Input parameters**	**Advantages**
Static BIV (sBIV)	Standardized	Measurements	Fixed cut-offs	Simple
Modified BIV (mBIV)	Empirical	Measurements	Fixed cut-offs	Time consensus, simple
Single-model outlier measurement detection (SMOM)	Statistical based	Measurements	Semi-dynamic^a^, based on the dataset	Population adjusted
Multi-model outlier measurement detection (MMOM)	Statistical and clustering based	Measurements	Semi-dynamic, based on the dataset	Group-adjusted
Clustering-based outlier trajectory (COT)	Clustering based	Trajectory	Dynamic, based on data size	Population adjusted
Multi-model outlier trajectory (MMOT)	Statistical and clustering based	Trajectory	Semi-dynamic, based on the dataset	Group-adjusted

^a^Combination of fixed thresholds (i.e. WHO) and dynamic thresholds or values derived from the dataset (including averages, number of clusters and so on)

Details for the sBIV, mBIV and MMOM methods were provided previously [28] and only key information is given here, for clarity.

- *Static BIV detection based on fixed outlier removal WHO cut-off values – sBIV:* This method is based on the static cut-offs established by the WHO for child growth [29]. Accordingly, cut-off values are established to categorize growth for various growth metrics including BMI, weight, and height/length for age z-scores. For example, zWFL growth <-5 SD or >5 SD is considered biologically implausible. In the context of this work, the sBIV method is applied in a cross-sectional manner, meaning per time-point of anthropometric measurement.
- *Modified BIV detection method - mBIV:* This method is a modified version of sBIV, described previously, as used within the TARGet Kids! cohort [12]. According to this method, a time-point measurement is flagged as a potential outlier according to the previous thresholds used in sBIV. However, in this case, prior and subsequent measurements of the subject to which the flagged measurement belongs are checked within 2 years. If no other measurement close to the flagged measurement, within 2 SD units, exists, then the flagged measurement is confirmed as an outlier. If such measurement exists, then this implies that the individual measurement is not an outlier but belongs to a particular subject/trajectory overall. The detailed process of mBIV is depicted in Supplementary Figure 1. More details on mBIV method and a comparison with sBIV are provided in Supplementary Section 2.
- *Single-model outlier measurement – SMOM:* The premise of the method, as it was outlined in mBIV, is that it may make more sense to seek outliers, not with respect to the individual according to fixed global thresholds, but with respect to the population that theindividual belongs in. To this end, we extracted an average trajectory from the entire studied population by calculating the average measurement per time-point. An individual’s measurements beyond ±2 SDs of the population mean for that time-point, were considered outliers. The ±2 SDs threshold is derived from a normal distribution, where 4.55% of the observations in a normally distributed dataset are expected to lie outside this range. This method is used as the second baseline for the time-point outlier detection.
- *Multi-model outlier measurement detection - MMOM:* The motivation behind this method is that prior research has shown that more than one distinct group might be present in growth data [34]. Under this assumption, we searched for outliers in the context of groups (or clusters) instead of considering the dataset as a single population or conserning specific individuals. For this method, we employed partitioning clustering (K-means) with Euclidean distance to detect clusters of growth trajectories [35]. The obtained clusters were then evaluated using a visual assessment of the clustering tendency (VAT), a tool that facilitates the visual assessment of cluster tendency in an unsupervised manner [36]. An average trajectory was derived per identified cluster and was based on the average measurement per time-point of all the trajectories in the same cluster. Outliers were flagged as follows: 1) the measurement of participants was averaged across each time-point separately for each cluster; 2) Using the average as a reference, we detected outliers that lay beyond ±2 SDs of the individual’s assigned cluster. The reason for choosing K-means is that partitioning algorithms tend to produce uniformly sized groups. For outlier detection, this is important because it avoids the creation of clusters that are too small or too large, where outliers do not have a significant impact.
- *Multi-model outlier trajectory detection – MMOT:* We employed the same multi-model principle as described above to detect outlier trajectories. Once again, we used K-means clustering with Euclidean distance to identify clusters and generated average models per cluster. Based on these, we calculated the mean and standard deviation of the residual sum-of-square (RSS) errors of all trajectories within a particular cluster. Finally, we considered as outliers the trajectories that had a greater than 2 SDs RSS error from the average model of their cluster. In practice, this method aims at finding trajectories that do not “fit” well in the cluster model, as represented by the average trajectory. Average representative trajectories were calculated for each cluster as described in the MMOM method, i.e., the trajectory with the average measurements per time-point of all subjects belonging to the same cluster.
- *Clustering-based outlier trajectory detection – COT:* A different approach to detecting outlier trajectories is evaluated based on hierarchical clustering (HC). Unlike partitioning algorithms, like K-means, HC tends to create unbalanced clusters with the potential of detecting some small clusters. In principle, these clusters should be further away from the population's main core, indicating potential outliers. This premise is further supported using the complete linkage criterion [34]. This linkage criterion uses an algorithm that classifies trajectories into clusters based on the shortest distance between their furthest data points. This linkage favours clusters of smaller diameter and higher in-cluster cohesion but does not necessarily optimise separation between clusters [35]. Thus, outliers should be isolated within small clusters, which was evaluated by determining the number of clusters (nc) using formula *(1)* and the total number of participants (n) as specified in [23]. This formula generates enough clusters so that the more unrelated clusters are kept disconnected and further away from the rest of the dataset.
  $$nc=\mathrm{max}(2,\frac{n}{10})$$

### Evaluation of detection methods on synthetic outliers

The first evaluation was based on the injected simulated outliers described previously. Injection created a controlled dataset and a set of known “true” outliers to compare against, providing an objective method to assess the performance of a detection method, given the perfect knowledge regarding the location of outliers. For per time-point detection, a true positive occurs when a method detects an outlier at the same time-point as the simulation approach injected it. A true positive for outlier trajectories occurs if a method detects an outlier trajectory that contains at least one injected outlier measurement. We also evaluated the performance when combined to assess the full potential of the proposed outlier detection methods. We performed pair-wise combinations for both per time-point and trajectory methods (i.e., mBIV-sBIV, mBIV-MMOM, MMOM-SMOM, and COT-MMOT). We examined if the applied combination improved the results of the individual methods.

### Impact of outlier detection methods on growth pattern analysis

To assess the impact of the outlier detection methods on the analysis of growth patterns, we conducted trajectory clustering upon two versions of the TARGet Kids! and CTX datasets: 1) original dataset, 2) original dataset with the addition of all synthetic outliers (type ALL) for each error density (Fig. 1). Growth patterns were detected using two clustering methods to take into account the effect of the method on cluster membership [34]: time series clustering (TSC) with HC, Euclidean distance and complete linkage [37], and latent class mixed models (LCMM) [38]. The natural cluster tendency of our datasets was assessed using the VAT tool [36] for TSC. For LCMM, the Bayesian Information Criterion was used to determine the optimal number of clusters and the trajectory shape. Group trajectories of obtained clusters were represented as smooth trending lines within each cluster using a locally estimated scatterplot smoothing (LOESS) method [39]. The clustering configurations obtained were compared based on their agreement, the percentage of subjects consistently grouped in the same clusters between clustering methods.

### Sensitivity analysis

We conducted two different sensitivity analyses; the first aimed to assess the impact of a different density of outliers. In our original experiments, synthetic outliers were randomly injected in 15% of the measurements, replacing the original measurements and resulting in one to two outlier data points for each child. For the first sensitivity analysis, we injected outliers in 30% of the children with four outliers each. This way we kept the same number of injected outliers but concentrated in fewer children. For the second sensitivity analysis, we modeled the population average trajectory using linear mixed effects models and examined whether the model fit is affected by outliers using root-mean-square error as a measure of model performance.

### Statistical analysis

For each method (sBIV, mBIV, SMOM, MMOM, COT, MMOT) and their combinations, performance was evaluated using sensitivity, specificity and precision in detecting the flagged outliers (sensitivity and specificity formulas available in Supplementary Section 3), and Cohen’s kappa statistic to test the agreement between the results of a method and the set of “true” injected outliers [40]. Independent samples t-test and analysis of variance (ANOVA) with Tukey’s test were used to compare the performance between methods adjusted for multiple comparisons. Analyses were conducted using R version 4.1.2 [41] and Stata 17 (StataCorp LP, College Station, TX, USA). The code artifacts can be found at https://github.com/Comelli-lab/detecting-outlier-measurements-and-trajectories-in-longitudinal-children-growth-data.

## Results

### Datasets characteristics and outlier injection

From the 2,342 infants originally considered from the TARGet Kids! dataset, 1,961 were excluded because a) they were born preterm (<=37 weeks) or very low birth weight (<1,500 g) (clinical criteria, *n*=1,127) and b) had at least one missing weight or length measurement (data quality criteria, *n*=734). Ultimately, we included 393 children with 3,144 measurements from the TARGet Kids! dataset. For the CTX dataset, from the 1,778 children considered initially, 221 were excluded because of the same clinical criteria, while 929 and 976 for zWA and zMUAC respectively, because of data quality criteria. Finally, we included 849 children with 7,641 measurements for zWA and 802 children with 7,218 measurements for zMUAC from the CTX dataset (Supplementary Table 1).

For the TARGet Kids! dataset, we generated 471 synthetic outlier measurements (157 for each of the three errors Type a, b, and c). A manual inspection before outlier injection revealed one additional BIV zWFL, which according to the synthetic outlier definition previously described, was included as a Type a error. In the end, for each error intensity, we created 4 outlier datasets containing outliers of Type a (*n*=158), Type b (*n*=157), Type c (*n*=157) or all combined error types (ALL, *n*=472). These outliers were considered “true” outliers and expected to be identified by the various detection methods. For outlier trajectories, we considered only the dataset with all types of errors (ALL) injected. For the subjects that had at least one outlier in this dataset when considering all types of errors (ALL), we injected outliers in 279 subjects with 1.6 outliers per subject on average.

For the CTX dataset and the zWA measurements, we generated 1,146 synthetic outliers (382 for each of the three errors Type a, b and c). For the subjects that had at least one injected outlier in this dataset, when considering all types of errors (ALL), we injected outliers in 648 subjects with a mean of 1.76 outliers per subject. For the zMUAC measurements, 1,083 synthetic outliers were generated (approximately 361 for each of the three types). For the subjects that had at least one outlier in this when considering all types of errors (ALL), we injected outliers in 615 subjects with 1.76 outliers per subject on average.

### Method performance evaluation for each outlier detection method

**Fig. 2:**
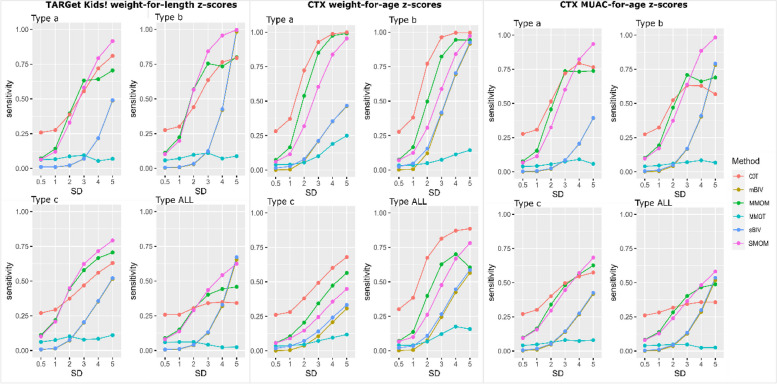
Sensitivity to detect outliers by detection method for each growth measure, error type and intensity. Abbreviations: TARGet Kids!, the applied research group for kids; CTX, the co-trimoxazole prophylaxis trial; zMUAC, mid-upper arm circumference-for-age z-scores; SD, error intensity as number of standard deviations injected to measurements; mBIV, modified method for biologically implausible values detection; sBIV; sBIV, static detection method for biologically implausible values based on fixed WHO cut-off values; MMOM, multi-model outlier measurement detection method; SMOM, single-model outlier measurement detection method; COT, clustering-based outlier trajectory detection method; MMOT, multi-model outlier trajectory detection method

Method performance per error types and densities are shown in detail in Fig. 2, Supplementary Tables 2, 3a, b and summarized below. Tables 2 and 3 show summaries of method performance. Since specificity levels were relatively high for all methods, due to the relatively low proportion of true outlier measurements, and sensitivity, precision and kappa follow similar trends, only sensitivity will be discussed here.

**Table 2:** Summary results on outlier method performance for measurement and trajectories

	**Sensitivity**				**Specificity**				**Precision**				**Kappa**			
	Min value	Experiment with min value	Max value	Experiment with max value	Min value	Experiment with min value	Max value	Experiment with max value	Min value	Experiment with min value	Max value	Experiment with max value	Min value	Experiment with min value	Max value	Experiment with max value
sBIV	0.2%	CTX, Type a, 0.5, zMUAC	99.6%	TK, Type b, 5, zWFL	98.8%	multiple settings	99.8%	multiple settings	2.4%	CTX, Type a, 0.5, zWA	98.5%	CTX, ALL, 5, zMUAC	0.002894	CTX, Type a, 0.5, zMUAC	0.967156	TK, Type b, 5, zWFL
mBIV	0.06%	CTX, Type a, 0.5, zWA	98.4%	TK, Type b, 5, zWFL	99.7%	multiple settings	99.9%	multiple settings	3.5%	CTX, Type a, 0.5, zWA	99.5%	CTX, ALL, 5, zMUAC	0.000735	CTX, Type a, 0.5, zWA	0.96498	TK, Type b, 5, zWFL
SMOM	5.6%	CTX, Type c, 0.5, zWA	99.6%	TK, Type b, 5, zWFL	94.9%	CTX, Type a, 0.5, zMUAC	99.9%	CTX, ALL, 5, zMUAC	2.4%	CTX, Type c, 0.5, zWA	99.4%	CTX, ALL, 5, zMUAC	0.006147	CTX, Type c, 0.5, zWA	0.930517	TK, Type b, 5, zWFL
MMOM	5.7%	CTX, Type c, 0.5, zWA	99.0%	CTX, Type a, 5, zWA	95.2%	CTX, Type c, 0.5, zWFL	99.8%	CTX, ALL, 5, zMUAC	2.4%	CTX, Type c, 0.5, zWA	98.7%	CTX, ALL, 5, zMUAC	0.005508	CTX, Type c, 0.5, zWA	0.717394	CTX, Type a, 4, zMUAC
COT	25.8%	TK, Type a, 0.5, zWFL	99.8%	CTX, Type a, 5, zWA	73.8%	CTX, Type b, 0.5, zWA	98.9%	CTX, ALL, 5, zMUAC	14.9%	CTX, Type c, 0.5, zWA	99.1%	CTX, ALL, 5, zMUAC	-0.00023	CTX, Type c, 0.5, zWA	0.906329	CTX, Type a, 5, zWA
MMOT	2.3%	TK, ALL, 4, zWFL	24.9%	CTX, Type a, 5, zWA	93.9%	TK, Type b, 0.5, zWFL	99.9%	TK, ALL, 5, zWFL	13.1%	CTX, Type b, 0.5, zWFL	99.8%	TK, ALL, 5, zWFL	-0.00769	CTX, Type b, 0.5, zWA	0.3212.91	CTX, Type a, 5, zWA

*Abbreviations*: *TARGet Kids!* The applied research group for kids, *zWFL* Weight-for-length z-scores, *zWA* weight-for-age z-scores, *CTX* The co-trimoxazole prophylaxis trial, *zMUAC* mid-upper arm circumference-for-age z-scores, *SD* Standard deviation, *mBIV* Modified method for biologically implausible values detection, *sBIV* Static WHO cut-off values for biologically implausible values detection method, *MMOM* Multi-model outlier measurement detection method, *SMOM* Single-model outlier measurement detection method, *COT* Clustering-based outlier trajectory detection method, *MMOT* Multi-model outlier trajectory detection method

The "setting" of minimum or maximum values corresponds to a tuple specifying the dataset (CTX or TARGet Kids!), the type of the error, the intensity of the error in SD, and the anthropometric measure

**Table 3:** Best method with respect to sensitivity, precision and kappa for each combination of intensity and type of error

Sensitivity								
	Type a		Type b		Type c		ALL	
SD	Method	Sensitivity	Method	Sensitivity	Method	Sensitivity	Method	Sensitivity
0.5	MMOM	7.66%	MMOM	11.22%	MMOM	11.17%	MMOM	8.87%
1	MMOM	16.46%	MMOM	22.41%	MMOM	21.88%	MMOM	15.02%
2	MMOM	53.78%	MMOM	56.84%	SMOM	44.89%	MMOM	39.83%
3	MMOM	85.08%	SMOM	84.24%	SMOM	62.27%	MMOM	62.77%
4	MMOM	97.49%	SMOM	95.64%	SMOM	71.58%	MMOM	70.08%
5	MMOM	99.08%	SMOM	99.68%	SMOM	79.22%	SMOM	78.09%
Precision								
	Type a		Type b		Type c		ALL	
SD	Method	Precision	Method	Precision	Method	Precision	Method	Precision
0.5	mBIV	17.29%	mBIV	25.13%	mBIV	18.15%	sBIV	35.00%
1	mBIV	32.21%	mBIV	52.02%	mBIV	75.18%	mBIV	85.77%
2	mBIV	86.19%	mBIV	93.42%	mBIV	94.10%	mBIV	96.78%
3	mBIV	95.71%	mBIV	97.83%	mBIV	97.26%	mBIV	98.52%
4	mBIV	97.89%	mBIV	99.00%	mBIV	98.49%	mBIV	99.29%
5	mBIV	98.94%	mBIV	99.44%	mBIV	99.05%	mBIV	99.58%
Kappa								
	Type a		Type b		Type c		ALL	
SD	Method	Kappa	Method	Kappa	Method	Kappa	Method	Kappa
0.5	MMOM	0.032371	MMOM	0.077747	MMOM	0.077343	MMOM	0.00075066
1	MMOM	0.110746	MMOM	0.199737	MMOM	0.193746	MMOM	0.17241
2	MMOM	0.406851	MMOM	0.530886	SMOM	0.43264	MMOM	0.391957
3	MMOM	0.676109	SMOM	0.767761	SMOM	0.621228	SMOM	0.550216
4	SMOM	0.751824	SMOM	0.879063	SMOM	0.728476	SMOM	0.659092
5	SMOM	0.866256	sBIV	0.967156	SMOM	0.810343	sBIV	0.767412

*Abbreviations*: *SD* Standard deviation, *mBIV* Modified method for biologically implausible values detection, *sBIV* Static WHO cut-off values for biologically implausible values detection method, *MMOM* Multi-model outlier measurement detection method, *SMOM* Single-model outlier measurement detection method

#### sBIV

Sensitivity to detect outliers ranged between 0.2-99.62%, precision 2.46-98.5%, kappa <0.01-0.96 and specificity between 98.8-99.86% in multiple combinations of data and injected outliers. The lowest values were observed at the lowest error intensity (0.5 SD), and vice versa for the highest error intensity (5 SD). Lower values for sensitivity, precision and Kappa were observed for Type a errors (because these errors may not always correspond to a BIV, especially for low error intensities). Higher values of sensitivity and Kappa were observed for Type b errors (the most extreme of the three types). Similarly, for precision in all (ALL) type of errors.

#### mBIV

Sensitivity ranged between 0.06-98.42%, precision between 3.50-99.58%, kappa between <0.01-0.96 and specificity between 99.71%-99.99% in multiple combinations. The observations regarding the types and intensities of errors were identical to those for sBIV, which is expected as the two methods share the same conceptual basis.

#### SMOM

Sensitivity ranged between 5.68-99.68%, precision between 2.46-99.40%, kappa between <0.01-0.93, and specificity between 94.95-99.94%. As in sBIV and mBIV, all metrics values varied in an error-intensity manner. However, low values for SMOM were observed for Type c (sensitivity, precision and Kappa) errors, which is expected as population-level models cannot capture deviations at the individual’s level. High values were observed for Type b (sensitivity, Kappa) and ALL types (precision) of errors.

#### MMOM

Sensitivity ranged between 5.72-99.08%, precision between 2.41-98.76%, kappa between <0.01-0.71, and specificity between 95.2-99.89%. Similarly to SMOM, the lowest values of MMOM were observed for the lowest error intensity and Type c errors, while unlike SMOM the highest values were observed for the highest error intensity, Type a (sensitivity, Kappa) and ALL types (precision) of errors. Type a errors may result in trajectory shape deviations, which may be easier to detect with group-based methods.

#### COT

Sensitivity ranged between 25.83-99.83% (CTX-zWA), precision between 14.93-99.12%, kappa ranged between <0-0.90, specificity between 73.82-98.93%. Concerning types of errors, while high values were fairly consistent between Type a (sensitivity, Kappa) and ALL types (specificity, precision), low values were observed for errors: Type a for sensitivity, Type b for specificity, Type c for precision and Kappa.

#### MMOT

Sensitivity ranged between 2.33-24.95%, precision between 13.18-99.88%, kappa ranged between <0-0.32, and specificity ranged between 93.99-99.99%. MMOT was the only method with a low value (sensitivity) to be observed for an error intensity other than 0.5 SD, which was 4 SD in this case. Type b error was the most observed one for low values (specificity, precision and Kappa), while Type a (sensitivity and Kappa) and ALL types (specificity, precision) were observed for high values.

Our analyses showed that mBIV and sBIV had generally similar performance across populations, growth measures, error types and intensities with their performance tending to increase for extreme errors and higher intensities (Fig. 2 and Supplementary Figure 2). Moreover, COT, MMOM and SMOM were constantly the best-performing methods by all three measures for all configurations. While MMOM was the best method for measurement outliers for CTX zWA error Type a,and b, c, for the rest of the configurations showed the best performance for error intensities below 2 or 3 SDs. For higher intensities, SMOM outperformed MMOM. MMOT was the worst method. Overall, all methods were affected by error type and intensity. More specifically, it can be observed in Fig. 2, all methods for all error types had sensitivity below 50% for low intensities (< 2 SDs). In addition, errors of Type c and ALL show low sensitivity across all methods and the two populations in comparison to Types a and b, which are more at the extreme end of spectrum of errors.

### Agreement between outlier detection methods

We next studied the agreement between outlier detection approaches for measurements and trajectories. We randomly selected one of the outlier simulations for ALL types of errors for both datasets and measurements (TARGet Kids!-zWA, CTX-zWA, CTX-zMUAC) and we evaluated the overlap between the outlier detection methods. When studying the agreement between two methods, we considered the intersection of true positives, how many of those were contributed by each of the combined methods, and the uniquely identified outliers by each of the combined methods. This analysis aimed to see if the combination can improve sensitivity and how much each combined method contributes to this improvement. We did the same analysis for false positives to study how much the combined methods contribute to the specificity.

Compared with all other outlier measurement detection methods (sBIV, MMOM and SMOM), mBIV did not contribute more true positives than its counterparts, except for within high-intensity errors (+/-5 SD). In this case, mBIV also contributed more false positives but few overall. However, sBIV always identified more true positives than mBIV, even for +/-5 SD errors. When comparing sBIV with MMOM and SMOM, this method also identified more true positives, for errors greater than +/-4 SD. However, the number of detected true positives was similar between sBIV and the other two methods for 4 SD as it was for 5 SD errors. When sBIV contributed more true positives, it also contributed more false positives when compared to any other method. When comparing MMOM and SMOM, the former contributed consistently more true positives than the latter for lower intensity errors (< 4 SD), but rarely more false positives. When comparing COT with MMOT to detect of outlier trajectories, COT detected more unique true positives than MMOT across all datasets, measures and error intensities, but it also contributed more unique false positives. The results of the agreement analysis were confirmed in two additional outlier simulations providing the same findings (data not shown).

### Performance of combinations of outlier detection methods

We next tested the performance of combining outlier detection methods in three random simulations that included ALL errors. Using the results of the agreement between the various methods, we focused on the pairs mBIV-MMOM, mBIV-SMOM, sBIV-MMOM, sBIV-SMOM, and MMOM-SMOM. These pairs were selected because both methods contributed similar amounts of true positives, thus their combination should increase their performance against the results of each method. Indeed, when studying sensitivity, the performance of the combination was always better or at least equal to one of the two combined methods. In fact, sensitivity increased up to 21.82% for the mBIV-MMOM pair. On the other hand, precision and specificity mostly decreased, since inevitably the combination also added false positives. However, the impact on specificity is minimal compared to the individual methods (Supplementary Table 4). Concerning outlier trajectory, we did not study the combination between COT and MMOT, because COT outperformed MMOT.

### Effect of outliers on clustering and growth pattern detection

**Fig. 3:**
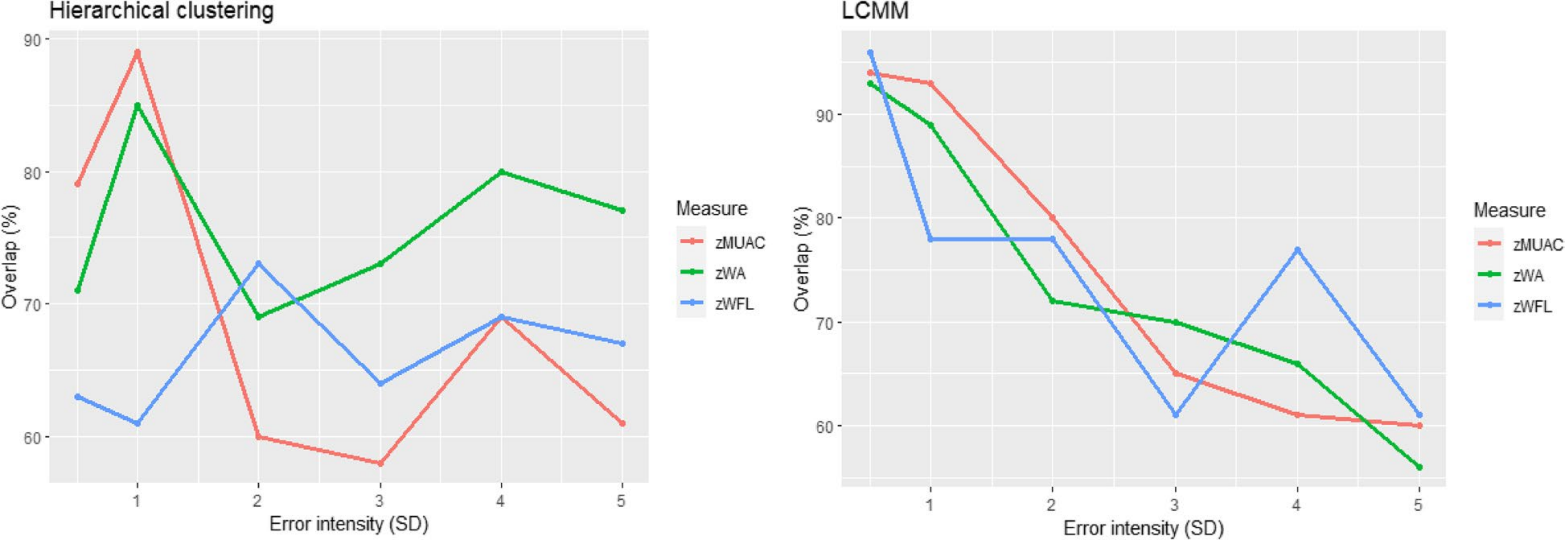
Clustering agreement for the 5 different error intensities using time series clustering (hierarchical clustering) and latent class mixed models (LCMM). Abbreviations: SD; standard deviation, LCMM; latent class mixed models

Supplementary Figure 3 presents clustering results obtained from the original TARGet Kids! and CTX datasets for all growth measures. Two distinct clusters were identified: TARGet Kids!-zWFL (cluster 1 (*n*=199) low normal, rapid increase and cluster 2 (*n*=194), normal, modest steady increase), CTX-zWA (cluster 1 (*n*=490), severe wasting, increase within abnormal levels, and cluster 2 (*n*=359), wasting, increase to normal levels) and CTX-zMUAC (cluster 1 (*n*=634), increase to normal levels, and cluster 2 (*n*=168), increase but within wasting). The same growth patterns were also identified with LCMM. Clustering overlap (agreement) varied between 57.9 -79.0% for all configurations (Fig. 3) showing that the presence of outliers caused cluster, and thus growth pattern misclassification, which increased with the increasing levels of error intensity Table 4.

**Table 4:** Key findings

1. Clustering-based outlier trajectory detection (COT) is a reliable method for outlier trajectory detection.
2. Combined detection methods for outlier measurements are preferred.
3. Some methods achieved >80% sensitivity for errors above 3 standard deviations
4. Model-based methods are reliable for errors of lower intensity.
5. Higher density favours outlier trajectory detection and model-based methods, but not time-sensitive methods.
6. Clustering and pattern analyses can be considerably affected by the presence of outliers.

### Sensitivity analyses

Our sensitivity analyses results are shown in Supplementary Tables 5 and 6. Studying the population average we found that the model accuracy was reduced, according to root mean square error, after the injection of the outliers in both populations in an error-intensity manner. Finally for the second part of our sensitivity analysis, the sensitivity of COT was considerably improved when outliers were in higher concentration in trajectories. On the contrary, COT performed worse at identifying trajectories with fewer outliers, potentially not outliers themselves. On the other hand, the sensitivity of mBIV was reduced, which is consistent with the mBIV design in which measurements within two years are not considered outliers. MMOM performed better detecting milder outliers, and MMOT showed low performance again. Error density affected method performance in a similar manner as in the main analysis.

## Discussion

Growth outliers need special considerations to be detected, eliminated, or otherwise addressed to minimize their impact on growth studies. We conducted a comprehensive assessment of types and intensities of outliers, detection methods, including detection of outlier trajectories, to crystallize these challenges. We conducted 432 different configurations to evaluate the performance of 6 different approaches to detect outliers of different types and intensities within growth measurements or trajectories in two different pediatric populations. We also assessed the impact of outliers on growth pattern detection and cluster assignment. We found that MMOM and SMOM were consistently better than mBIV and sBIV in terms of sensitivity across populations, error types and levels. This confirms that methods need to be sensitive enough to detect both mild and extreme outliers. This is in agreement with our preliminary work in which MMOM outperformed both sBIV and mBIV and had a similar performance to SMOM, although error intensity was not investigated [28].

Our results showed that the model-based approaches constantly showed relatively high performance, even for low error intensity levels (<3 SDs), and were at least as accurate as BIV methods, if not better. This may indicate the model-based approaches are superior to BIV-based approaches. Both types of detection methods improved their accuracy as the error intensity increased, but the increase for model-based approaches was less prominent when the error intensity increased (>3 SDs). Between SMOM and MMOM, the former was consistently better than the latter except for zWFL measures in the CTX dataset. MMOM performed better when the error intensity was low (<2 SDs), but SMOM became more accurate for higher error intensities (>3 SDs). One possible justification for the difference between SMOM and MMOM is that clustering-based approaches, especially those using partitioning, are more sensitive to outliers, because they affect the identified clusters, as our sensitivity analysis showed. Overall, we can argue that SMOM remains a reliable outlier detection method for measurements, and also has simplicity. While mBIV succeeded in finding BIVs represented by Type b (extreme) errors, this was also the case for the MMOM method, which may indicate that the latter can be used to detect a broader spectrum of outliers, including all of those identified by mBIV. Thus, MMOM may be considered a more holistic approach to identifying a broader spectrum of single outliers.

We also compared our method’s sensitivity to the conditional growth percentiles [19] outlier detection method.

In addition, our experimentation on the performance of combinations of outlier detection methods showed that no combination outperformed MMOM when applied alone, suggesting that MMOM may be sufficient in detecting all types of synthetic outliers. Similarly, COT outperforms the combination approach of MMOT and COT, suggesting that it is also sufficient in detecting all types of synthetic outliers within trajectories. Our analyses also showed that the performance of outlier detection methods differed where most could detect Type b errors (extreme outliers) but varied in their capacity to flag more subtle (mild) or complex trajectory cases. COT effectively detected both Type a and b errors and had the highest sensitivity in detecting trajectories with frequent or a series of odd measurements. These trajectories can indicate a unique subgroup within the dataset (e.g., children with specific diseases or from a particular ethnicity or low-resource setting). Cluster-based cleaning approach depends on selecting a suitable number of groups to model, and this decision should take into account the well-known principle: “The more and smaller clusters we have, the more cohesive they are (smaller diameter) and the farther apart from each other they are [22].”

We also showed the effect of outliers in growth pattern detection using two different clustering approaches, time series clustering and LCMM. Regardless of the grouping method, we can confirm that outliers affect grouping by at least 57%. This means that a big part of the population will be assigned to the wrong growth pattern, which can affect associations with health outcomes. Furthermore, we found that not only subjects had outliers injected in them but also “clean” subjects that moved between patterns. This shows that any outlier detection should be performed before the analysis because outliers affect not only the results but the process as a whole. This observation is aligned with data analyses models, such as the cross-industry process for data mining model, which serves as the base for a data science process, and propose data cleaning as part of a more extensive "Data Preparation" phase which proceeds modelling [42]. Finally, our sensitivity analyses showed that the SD threshold could also impact outlier method performance. This finding is logical for two reasons. First, the lower the threshold is, the more outliers will be detected by the method, as fewer measurements or trajectories will remain close enough to the average to avoid detection. Second, the fewer outliers found beyond a high SD threshold, the higher the chances that they will be outliers (true positives), implying increased precision. While the finding is reasonable, the variation remains, which can be a potential design problem for studies that involve outlier detection. In this case, if a threshold is not intuitive or cannot be supported clinically, one may instead rely on other methods that do not require an SD threshold, like some of those proposed within this work (i.e. mBIV and COT).

Although SMOM, MMOM and COT performance varied per configuration, their sensitivity is among the highest in the literature for outlier detection in pediatric growth data. In the work of Shi et al, 2018, [18] sensitivity varied between 10.7-14.1% for the Jackknife residuals method and between 0.1-0.2% for the conditional growth percentile method in the same population [19]. Other methods, including exponentially weighted moving average standard deviation scores and regression-based weight change models also showed low sensitivity (<19%) [43]. Our work showed that model-based approaches have the best performance for detecting outlier measurements. This is in agreement with the work of Woolley et al. [44], where the non-linear mixed-effects model cut-off had the highest sensitivity, which was also improved with a decision-making algorithm. This decision-making algorithm modified or deleted flagged measurements, which however was not applicable in this study. Finally, our study agrees with our previous findings that the static and modified for longitudinal measurements WHO cut-offs have low performance [18]. In fact, the WHO growth standards were intentionally developed using populations with community children whose growth is not representative of disadvantaged children, including those with severe malnutrition [15, 45, 46].

To the best of our knowledge, this is the first work that applies a clustering-based approach to flag growth outliers of different types and intensities and at the same time to propose a method for detecting outlier trajectories, one of the most popular tools for studying and representing growth. The study comprehensively compares several outlier detection methods and their configurations on two real-world datasets, outlining their strengths and limitations and discussing the challenges of outlier detection for children's growth data. We conducted extensive experimentation focusing on outliers’ characteristics, error types and intensities in two different populations with CTX, including a unique population rarely studied in such a context. This work has also limitations. First, we used synthetic outliers, while future works could use “wild” outliers that are identified and corrected in clinical settings. Second, the ALL type of error amounts to 15% of the total measurements as it includes all the other types of errors. To alleviate this limitation we compared the algorithms under the same conditions. We also tried to secure compatibility between methods by excluding children with missing measurements in both datasets which, however, reduced the number of eligible participants in this study. Our work aims to construct a framework for detecting outliers in longitudinal growth data, allowing our methods to be extended, adapted and applied to datasets with different properties, such as missing measurements or trajectories of varying lengths. By using distance metrics that allow for missing data, like Fréchet's distance [47], clustering configurations can be adapted and therefore study all participants in cohorts.

## Conclusion

In conclusion, model-based approaches that detect outliers assuming multiple groups in the sample show the best performance. Using clustering to detect outliers is a reliable method. Finally, the type of the outlier can affect performance and have an important impact on growth pattern detection. Since outlier detection is a process that needs to precede modelling along with treating missing values and correcting data input errors [42], we believe that our methods can have practical applications for children growth analyses studies

### Supplementary Information

**Additional file 1:** **Supplemental Figure 1.** Flow-chart describing data cleaning procedures using the modified BIV detection method (mBIV). Cleaning of age- and sex-standardized WHO weight-for-length z-scores (zWFL) outlier measurements is used as an example to demonstrate the method.* Abbreviations: RA, research assistants; SD, standard deviation*. **Supplemental Figure 2.** Methods precision (panel A) and kappa (panel B) for all error types and intensities. **Supplemental Figure 3.** TARGet Kids! zWFL and CTX zWA and zMUAC clusters obtained via hierarchical clustering using original (without artificial outliers) data. **Supplemental Section 1.** Definition of error type (how the error is added to the measurement). **Supplemental Section 2.** Difference between static BIV detection based on fixed outlier removal WHO cut-off values (sBIV) and the modified BIV detection method (mBIV). **Supplemental Section 3.**Formula definitions as in (1-3). **Supplemental Table 1.** Summary of anthropometric measurements available for the TARGet Kids! and the CTX trial dataset. **Supplemental Table 2.** Summary of results from 100 simulation experiments of outlier detection methods applied on the TARGet Kids! Dataset. Expressed as mean (SD) for weight-for-length z-scores. **Supplemental Table 3a.** Summary of results from 100 simulation experiments of outlier detection methods applied on the CTX Dataset. Expressed as mean (SD) for MUAC z-scores. **Supplemental Table 3b.** Summary of results from 100 simulation experiments of outlier detection methods applied on the CTX Dataset. Expressed as mean (SD) for weight-for age z-scores. **Supplemental Table 4.** Sensitivity, specificity, precision and kappa per growth measure and dataset when combing outlier measurement detection methods (Method A and B). **Supplemental Table 5.** Model fitting parameters for the population average trajectory for the original dataset and for the dataset with outliers of 6 different intensities. **Supplemental Table 6.** Summary of sensitivity results with 4 outliers per trajectory applied on the TARGet Kids! Dataset.

## Data Availability

Applications to access these data can be made by completing a data request application form available through the study investigators. The co-trimoxazole trial growth data are available at: https://dataverse.harvard.edu/dataset.xhtml?persistentId=doi:10.7910/DVN/XG8KDS

## Abbreviations

ALL: All types
ANOVA: Analysis of variance
BIV: Biologically implausible value
COT: Clustering outlier trajectory detection method
CTX: co-trimoxazole
HC: Hierarchical clustering
LCMM: Latent class mixed models
mBIV: Modified method for biologically implausible values
MMOM: Multi-model outlier measurement detection method
MMOT: Multi-model outlier trajectory detection method
MUAC: Mid-upper arm circumference
RSS: Residual sum-of-squares
SD: Standard deviation
SMOM: Single-model outlier measurement detection method
TARGet Kids!: The applied research group for kids
TSC: Time series clustering
VAT: Visual assessment of the clustering tendency
WHO: World health organization
zBIV: Static biologically implausible value detection method
zMUAC: Age- and sex-standardized values for mid-upper arm circumference
zWA: Age- and sex-standardized values for weight
zWFL: Age- and sex-standardized weight-for-length
