# Supplementary material for: New approaches and technical considerations in detecting outlier measurements and trajectories in longitudinal children growth data
Source: BMC Med Res Methodol. 2023 Oct 13;23:232. doi: 10.1186/s12874-023-02045-w (PMC10576311; doi:10.1186/s12874-023-02045-w)
Supplement: Supplementary file 1 — Additional file 1: Supplemental Figure 1. Flow-chart describing data cleaning procedures using the modified BIV detection method (mBIV). Cleaning of age- and sex-standardized WHO weight-for-length z-scores (zWFL) outlier measurements is used as an example to demonstrate the method. Abbreviations: RA, research assistants; SD, standard deviation. Supplemental Figure 2. Methods precision (panel A) and kappa (panel B) for all error types and intensities. Supplemental Figure 3. TARGet Kids! zWFL and CTX zWA and zMUAC clusters obtained via hierarchical clustering using original (without artificial outliers) data. Supplemental Section 1. Definition of error type (how the error is added to the measurement). Supplemental Section 2. Difference between static BIV detection based on fixed outlier removal WHO cut-off values (sBIV) and the modified BIV detection method (mBIV). Supplemental Section 3.Formula definitions as in (1-3). Supplemental Table 1. Summary of anthropometric measurements available for the TARGet Kids! and the CTX trial dataset. Supplemental Table 2. Summary of results from 100 simulation experiments of outlier detection methods applied on the TARGet Kids! Dataset. Expressed as mean (SD) for weight-for-length z-scores. Supplemental Table 3a. Summary of results from 100 simulation experiments of outlier detection methods applied on the CTX Dataset. Expressed as mean (SD) for MUAC z-scores. Supplemental Table 3b. Summary of results from 100 simulation experiments of outlier detection methods applied on the CTX Dataset. Expressed as mean (SD) for weight-for age z-scores. Supplemental Table 4. Sensitivity, specificity, precision and kappa per growth measure and dataset when combing outlier measurement detection methods (Method A and B). Supplemental Table 5. Model fitting parameters for the population average trajectory for the original dataset and for the dataset with outliers of 6 different intensities. Supplemental Table 6. Summary of sensitivity results with 4 [file 12874_2023_2045_MOESM1_ESM.docx]

**Supplemental Figure 1:** Flow-chart describing data cleaning procedures using the modified BIV detection method (mBIV). Cleaning of age- and sex-standardized WHO weight-for-length z-scores (zWFL) outlier measurements is used as an example to demonstrate the method. *Abbreviations: RA, research assistants; SD, standard deviation*


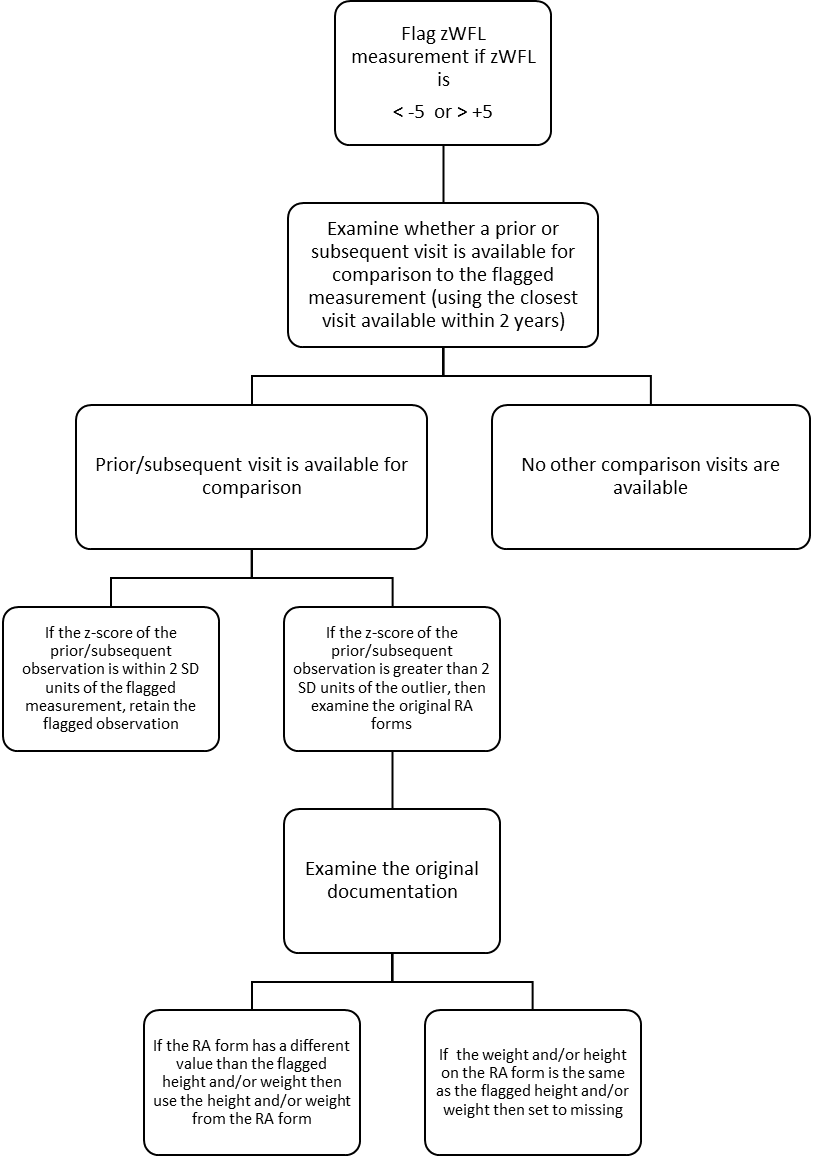


**Supplemental Figure 2:** Methods precision (panel A) and kappa (panel B) for all error types and intensities.


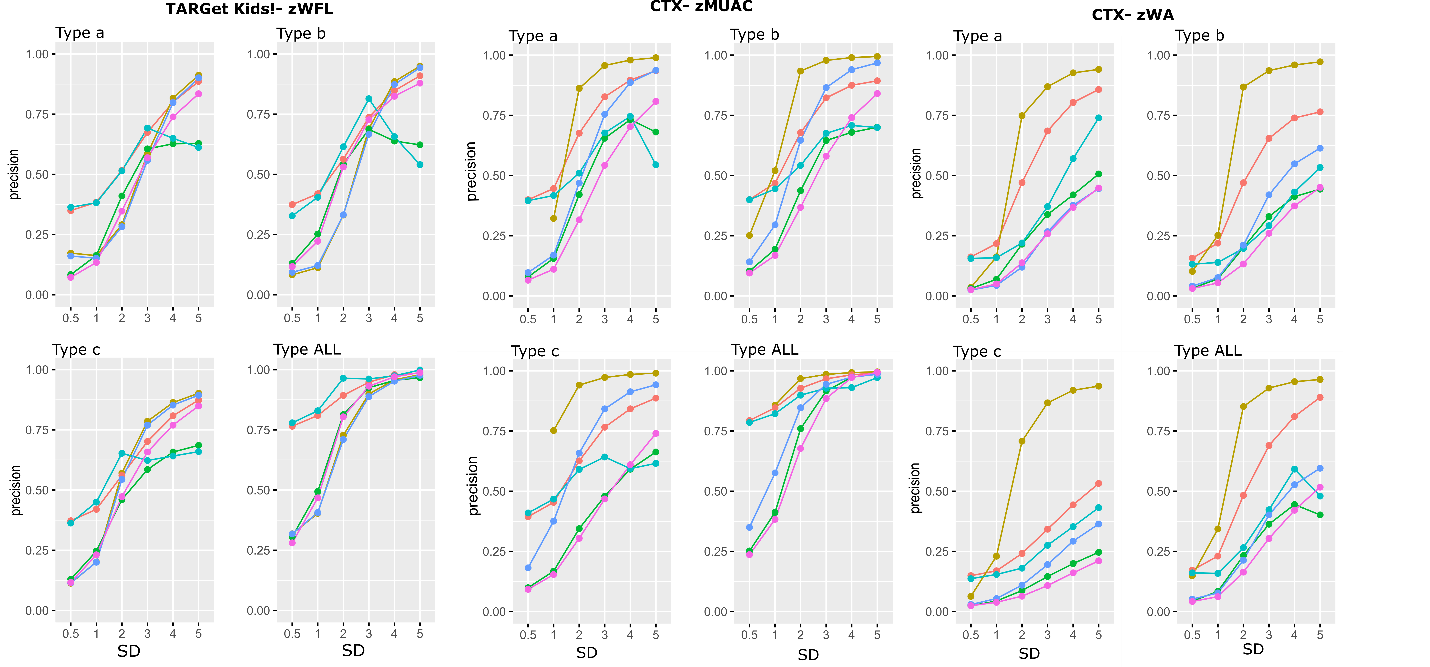


A


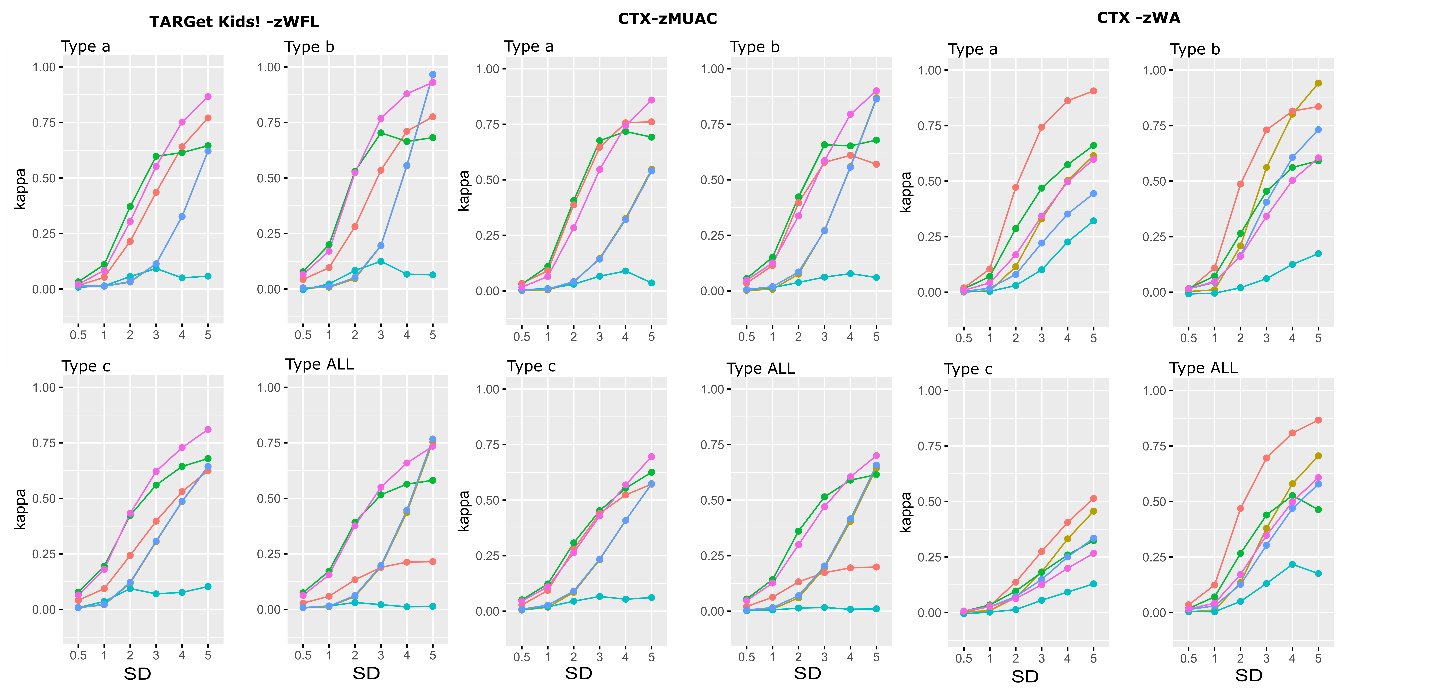


B


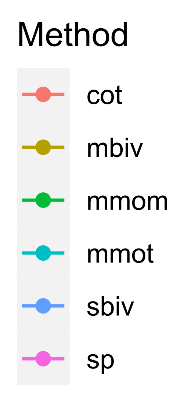


***Abbreviations:*** *TARGet Kids!, the applied research group for kids; CTX, the co-trimoxazole prophylaxis trial; zMUAC, mid-upper arm circumference-for-age z-scores; zWA, weight-for-age z-scores; SD, standard deviation; mBIV, modified method for biologically implausible values detection; sBIV, static WHO cut-off values for biologically implausible values detection method; MMOM, multi-model outlier measurement detection method; SMOM, single-model outlier measurement detection method; COT, clustering-based outlier trajectory detection method;* *MMOT, multi-model outlier trajectory detection method.*


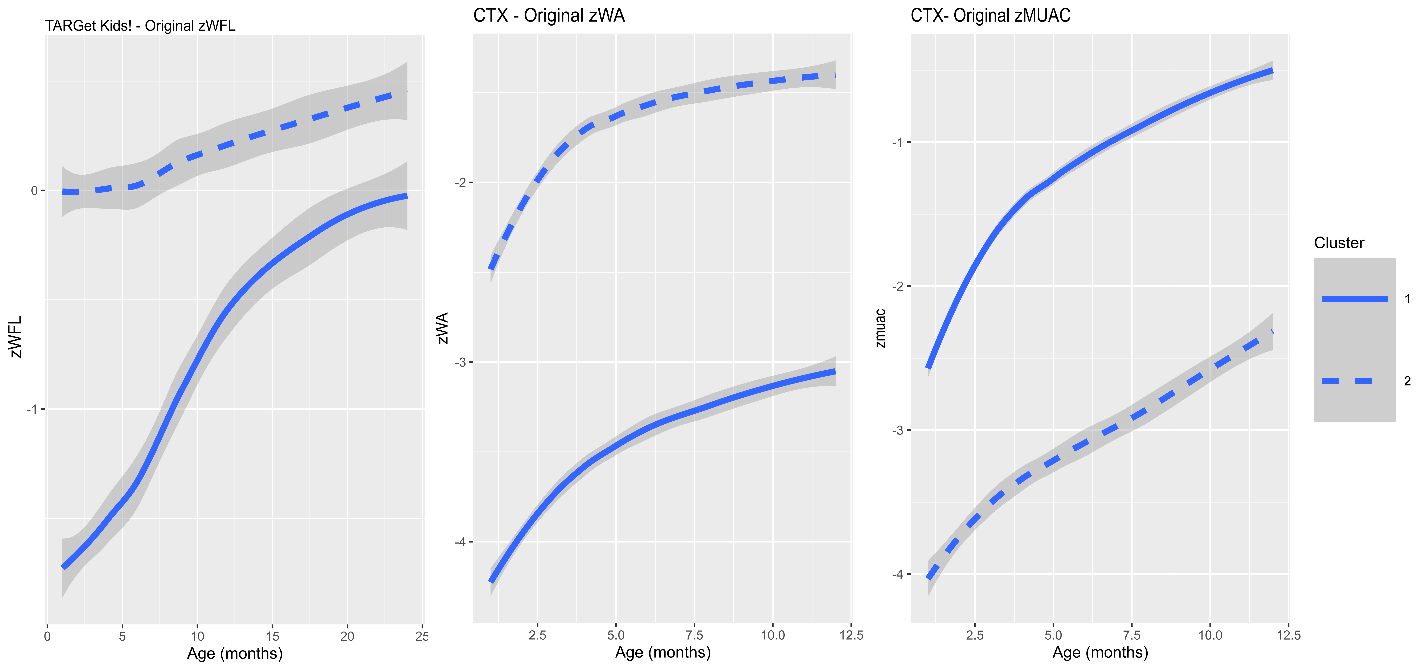
**Supplemental Figure 3:** TARGet Kids! zWFL and CTX zWA and zMUAC clusters obtained via hierarchical clustering using original (without artificial outliers) data.

**Supplemental Section 1:** Definition of error type (how the error is added to the measurement).

**Process**

Synthetic errors randomly injected to both datasets to create global (i.e., which exceed the WHO standards) and contextual (i.e., within the context of an individual child) outliers. A number of measurements of the dataset, corresponding roughly to the 5% of the total dataset, are randomly selected to be modified as outliers by a synthetically generated error. In addition, we experimented with 6 different levels of intensity for the injected errors ranging from 0.5 to 5 standard deviations (SDs). We create three types of errors described in detail next. Outliers were injected in 5% of the dataset measurements for each type of error, resulting in 15% outliers for ALL types. The synthetic outliers were flagged and used as the “gold standard” for each of the six methods of evaluation. Each outlier injection process was repeated 100 times and the overall performance of the detection methods was evaluated as an average over the 100 different datasets. Eventually, we created 24 different datasets (4 types of errors and 6 levels of intensities) for each population dataset (1 for TARGet Kids! and 2 for CTX). A total of 7,200 datasets with outliers were studied. With respect to outlier trajectories, if at least one measurement of a trajectory was modified by an injected error, this trajectory is considered an outlier trajectory. This definition may be too limited, which is why we conducted a sensitivity analysis to assess the density of errors in outlier trajectories.

**Type a (moderate to extreme)**: Given the selected intensity (0.5-5 SDs), a random sign (positive or negative) is assigned to the error. For example, if the intensity level is 3 SD and the random sign is (+) the synthetic error is +3 SD. Next, the synthetic error is added to the randomly selected measurement. If the measurement corresponds to a negative z-score, e.g., -2, the resulting outlier will be +1, which is considered a moderate outlier. If the measurement is +2, the resulting outlier will be +5, which is considered a more extreme outlier.

**Type b (extreme)**: In this case, the error with the corresponding level of intensity is added to the absolute value of the random measurement. For example, if the measurement is -2 z-scores and the error is 3, then the resulting outlier will be -5. This will always result in an extreme outlier, potentially crossing the threshold for biologically implausible values.

**Type c (local)**: In Types a and b, the generate error was added as-is to the measurement. In Type c, we always calculate the average and the standard deviation of the measurements of specific subject. Then, the intensity of the error is multiplied as a weight and the result is added to the random measurement as in the errors of Type b. For example, if the intensity is 4, the random measurement is -2 and the standard deviation of the subject, to which the random measurement belongs, is 0.25, the error is 1 and the resulting outlier is -(2+1) = -3. This type of errors results in extreme errors, but only locally to the subject and not always to biologically implausible values.

**ALL (combination of all types)**: As a final analysis, we considered the combination of all types of errors in the same dataset. One important note here is that, while in each individual errors, the outliers accounted for 5% of the dataset, the combination was additive and resulted in 15% of the measurements being outliers. The dataset with ALL types included all types of errors. While this potentially skews the accuracy of the studied method, it was also important to consider the impact of each type of error when considering all errors. Thus, each type is still represented by a 5% instead of a mere 1.5% if we were to maintain the 5% outlier ratio.

**Supplemental Section 2:** Difference between static BIV detection based on fixed outlier removal WHO cut-off values (sBIV) and the modified BIV detection method (mBIV).

sBIV detects a measurement as outlier if it lies outside of +/- 5SD of the z-score measurement. mBIV flags such a measurement as a potential outlier, but then further confirms it as such if there is no measurement that is close to the flagged one (within 2 SDs) within a period of 2 years. If there is, then the flagged measurement is not considered an outlier, but a regular measurement within a subject with a potential growth issue. sBIV does not make this differentiation with the potential of flagging measurements in such subjects as outliers. The 2 years interval has been derived empirically by the TARGet Kids! Cohort.

**Supplemental Section 3:** Formula definitions as in (1-3).

|  | **Reference** | |
| --- | --- | --- |
| **Predicted** | Outlier | No outlier |
| Outlier | TP | FP |
| No outlier | FN | TN |

The formulas used here are:

- Sensitivity = TP / (TP + FN)
- Specificity = TN / (FP + TN)
- Precision = TP / (TP + FP)

*Abbreviations: TP, true positive; TN, true negative; FP, false positive; FN, false negative.*

**Supplemental Table 1:** Summary of anthropometric measurements available for the TARGet Kids! and the CTX trial dataset.

|  | TARGet Kids! dataset (n=393) | | | CTX dataset (n=849) | | | | |
| --- | --- | --- | --- | --- | --- | --- | --- | --- |
|  | Weight (Kg) | Length (cm) | zWFL | MUAC (mm) | Weight (Kg) | Height (cm) | zWA | zMUAC |
| Total number of the included measurements | 3,144 | 3,144 | 3,144 | 12,170 | 15,993 | 10,397 | 11,738 | 10,047 |
| Mean (SD) of the included measurements | 10.10 (120.27)*/  8.31 (2.84)** | 70.73 (16.62 )*/  71.16 (11.08)** | -0.424 (1.282)* | 12.53 (1.489) | 5.81*(1.58) | 73.19 (7.641)* | -2.848 (1.343)* | -1.824 (1.369)* |
| Median (range) of the included measurements | 8.16 (2.31-16.6)** | 71.16 (45-102.5)** | -0.38 (-7.1 – 4.81)* | 12.5 (6.6-17.8) | 5.74 (1.76-14.80)* | 73.00 (46.20-109.40)* | -2.81 (-7.69 1.58)* | -1.77 (-7.81 – 2.63)* |
| NAs | 4,712 | 4,712 | 4,712 | 3,832 | 9 | 3,616 | 4,264 | 5,955 |
|  |  |  |  |  |  |  |  |  |
| *Abbreviations: TARGet Kids!, The Applied Research Group for Kids; NA, not available; SD, standard deviation;* *CTX, the co-trimoxazole prophylaxis trial; zWFL, weight-for-length z-scores; zWA, weight-for-age z-scores; zMUAC, mid-upper arm circumference z-scores.*  **Available measurments after excluding preterm and very low birth weight infants **Available measurments after excluding preterm and very low birth weight infants and children with missing data.* | | | | | | | | |

**Supplemental Table 2:** Summary of results from 100 simulation experiments of outlier detection methods applied on the TARGet Kids! Dataset. Expressed as mean (SD) for weight-for-length z-scores.

| **Outlier type** | **Detection method** | **SD error** | **Sensitivity** | **Specificity** | **Precision** | **Kappa** |
| --- | --- | --- | --- | --- | --- | --- |
|  |  |  |  |  |  |  |
| **Type a** | sBIV | 0.5 | 1.05% (0.48)^3^ | 99.71% (0.02)^3^ | 16.09% (6.98)^6^ | 0.0137 (0.0090) |
|  |  | 1 | 1.01% (0.55)^3^ | 99.71% (0.02)^3^ | 15.16% (7.14)^5^ | 0.0128 (0.0099) |
|  |  | 2 | 2.19% (0.78)^3^ | 99.71% (0.02)^3^ | 28.29% (7.71)^2^ | 0.0338 (0.0137) |
|  |  | 3 | 6.95% (1.68)^3^ | 99.72% (0.02)^3^ | 55.83% (7.01)^8^ | 0.1137 (0.0273) |
|  |  | 4 | 21.73% (2.90)^3^ | 99.71% (0.03)^3^ | 79.81% (2.75)^1^ | 0.3266 (0.0376) |
|  |  | 5 | 49.1% (4.34)^3^ | 99.72% (0.02)^3^ | 90.10% (1.08)^1^ | 0.6214 (0.0392) |
|  | mBIV | 0.5 | 1.02% (0.48)^3^ | 99.75% (0.02)^3^ | 17.29% (7.50)^6^ | 0.0138 (0.0088) |
|  |  | 1 | 0.97% (0.51)^3^ | 99.74% (0.02)^3^ | 16.26% (7.36)^5^ | 0.0128 (0.0093) |
|  |  | 2 | 2.02% (0.74)^3^ | 99.75% (0.02)^3^ | 29.07% (8.46)^2^ | 0.0316 (0.0137) |
|  |  | 3 | 6.84% (1.62)^3^ | 99.75% (0.03)^3^ | 58.55% (7.13)^8^ | 0.1132 (0.0266) |
|  |  | 4 | 21.62% (2.88)^3^ | 99.75% (0.03)^3^ | 81.69% (2.72)^1^ | 0.3272 (0.0377) |
|  |  | 5 | 48.79% (4.24)^3^ | 99.75% (0.03)^3^ | 91.18% (1.23)^1^ | 0.6216 (0.0386) |
|  | SMOM | 0.5 | 6.41% (1.84)^3^ | 95.67 % (0.10)^3^ | 7.23% (2.01)^6^ | 0.0218 (0.1099) |
|  |  | 1 | 12.04% (2.58)^3^ | 95.90% (0.13)^3^ | 13.43% (2.74)^5^ | 0.0835 (0.0276) |
|  |  | 2 | 32.96% (3.64)^3^ | 96.73% (0.16)^3^ | 34.76% (3.28)^2^ | 0.3043 (0.0358) |
|  |  | 3 | 58.18% (3.62)^3^ | 97.67% (0.12)^3^ | 56.91% (2.42)^8^ | 0.5526 (0.0305) |
|  |  | 4 | 79.32% (2.93)^3^ | 98.51% (0.12)^3^ | 73.85% (2.01)^1^ | 0.7518 (0.0236) |
|  |  | 5 | 91.58% (1.96)^3^ | 99.04% (0.10)^3^ | 83.48% (1.39)^1^ | 0.8662 (0.0133) |
|  | MMOM | 0.5 | 7.2% (1.90)^3^ | 95.82% (0.13)^3^ | 8.35% (2.16)^6^ | 0.0324 (0.0210) |
|  |  | 1 | 14.16% (3.09)^3^ | 96.18% (0.16)^3^ | 16.35% (3.31)^5^ | 0.1103 (0.0331) |
|  |  | 2 | 39.52% (3.79)^3^ | 96.99% (0.17)^3^ | 41.02% (3.22)^2^ | 0.3714 (0.0360) |
|  |  | 3 | 63.23% (6.44)^3^ | 97.83% (0.25)^3^ | 60.58% (4.71)^8^ | 0.5978 (0.0569) |
|  |  | 4 | 64.15% (4.50)^3^ | 97.98% (0.23)^3^ | 62.71% (3.58)^1^ | 0.6143 (0.0394) |
|  |  | 5 | 70.50% (3.44)^3^ | 97.79% (0.23)^3^ | 62.87% (2.90)^1^ | 0.6455 (0.0296) |
|  | COT | 0.5 | 25.83% (4.83)^1^ | 75.64% (3.49)^1^ | 34.96% (4.51)^7^ | 0.0156 (0.0518) |
|  |  | 1 | 27.57% (4.75)^1^ | 77.36% (3.94)^1^ | 38.42% (5.19)^7^ | 0.0530 (0.0558) |
|  |  | 2 | 38.80% (6.15)^1^ | 81.38% (4.33)^1^ | 51.70% (5.21)^7^ | 0.2146 (0.0570) |
|  |  | 3 | 55.57% (6.92)^1^ | 86.22% (3.03)^1^ | 67.42% (4.63)^7^ | 0.4358 (0.0627) |
|  |  | 4 | 71.93% (6.42)^1^ | 90.64% (2.68)^1^ | 80.03% (4.47)^1^ | 0.6406 (0.0585) |
|  |  | 5 | 80.99% (8.25)^1^ | 94.57% (2.20)^1^ | 88.60% (4.15)^1^ | 0.7703 (0.0739) |
|  | MMOT | 0.5 | 6.22% (1.49)^1^ | 94.45% (0.83)^1^ | 36.34% (8.72)^7^ | 0.0085 (0.0283) |
|  |  | 1 | 6.62% (1.73)^1^ | 94.53% (0.93)^1^ | 38.21% (9.86)^7^ | 0.0144 (0.0322) |
|  |  | 2 | 8.60% (1.72)^1^ | 95.89% (0.82)^1^ | 51.45% (8.92)^7^ | 0.0564 (0.0274) |
|  |  | 3 | 9.42% (2.15)^1^ | 97.88% (0.73)^1^ | 69.23% (9.65)^7^ | 0.0925 (0.0298) |
|  |  | 4 | 5.36% (2.25)^1^ | 98.54% (0.67)^1^ | 64.98% (13.76)^1^ | 0.0502 (0.0316) |
|  |  | 5 | 6.89% (1.66)^1^ | 97.69% (0.87)^1^ | 61.15% (10.55)^1^ | 0.0584 (0.0238) |
| **Type b** | sBIV | 0.5 | 0.64% (0.58)^3^ | 99.68% (0.02)^3^ | 9.34% (8.21)^2^ | 0.0058 (0.0106) |
|  |  | 1 | 0.89% (0.81)^3^ | 99.68% (0.02)^3^ | 12.13% (10.20)^6^ | 0.0102 (0.0147) |
|  |  | 2 | 3.28% (1.72)^3^ | 99.68% (0.02)^3^ | 33.19% (12.65)^6^ | 0.0515 (0.0295) |
|  |  | 3 | 12.40% (2.61)^3^ | 99.68% (0.03)^3^ | 66.68% (4.97)^9^ | 0.1962 (0.0383) |
|  |  | 4 | 42.57% (4.91)^3^ | 99.68% (0.02)^3^ | 87.35% (1.52)^1^ | 0.5568 (0.0476) |
|  |  | 5 | 99.62% (0.50)^4^ | 99.68% (0.02)^3^ | 94.29% (0.39)^3^ | 0.9672 (0.0036) |
|  | mBIV | 0.5 | 0.51% (0.51)^3^ | 99.71% (0.02)^3^ | 8.27% (8.15)^2^ | 0.0040 (0.0095) |
|  |  | 1 | 0.75% (0.75)^3^ | 99.71% (0.02)^3^ | 11.26% (10.30)^6^ | 0.0082 (0.0136) |
|  |  | 2 | 2.94% (1.59)^3^ | 99.71% (0.02)^3^ | 33.11% (12.99)^6^ | 0.0466 (0.0275) |
|  |  | 3 | 12.20% (2.62)^3^ | 99.72% (0.03)^3^ | 68.95% (4.82)^9^ | 0.1947 (0.0386) |
|  |  | 4 | 41.99% (4.76)^3^ | 99.72% (0.03)^3^ | 88.55% (1.60)^1^ | 0.5542 (0.0468) |
|  |  | 5 | 98.42% (1.03)^4^ | 99.73% (0.03)^3^ | 95.00% (0.53)^3^ | 0.9650 (0.0061) |
|  | SMOM | 0.5 | 10.27% (2.26)^3^ | 95.94% (0.10)^3^ | 11.72% (2.45)^2^ | 0.0659 (0.0244) |
|  |  | 1 | 19.82% (2.54)^3^ | 96.36% (0.12)^3^ | 22.23% (2.53)^6^ | 0.1705 (0.0263) |
|  |  | 2 | 56.59% (3.36)^3^ | 97.37% (0.12)^3^ | 53.08% (2.12)^6^ | 0.5231 (0.0272) |
|  |  | 3 | 84.24% (2.85)^3^ | 98.34% (0.11)^3^ | 72.69% (1.60)^9^ | 0.7678 (0.0197) |
|  |  | 4 | 95.64% (1.95)^3^ | 98.93% (0.09)^3^ | 82.46% (1.20)^1^ | 0.8791 (0.0126) |
|  |  | 5 | 99.68% (0.75)^4^ | 99.28% (0.09)^3^ | 87.91% (1.31)^3^ | 0.9305 (0.0080) |
|  | MMOM | 0.5 | 11.22% (2.79)^3^ | 96.06% (0.14)^3^ | 12.98% (3.00)^2^ | 0.0777 (0.0299) |
|  |  | 1 | 22.41% (3.11)^3^ | 96.52% (0.13)^3^ | 25.23% (3.00)^6^ | 0.1997 (0.0317) |
|  |  | 2 | 56.84% (3.59)^3^ | 97.48% (0.15)^3^ | 54.22% (2.52)^6^ | 0.5309 (0.0303) |
|  |  | 3 | 75.45% (8.14)^3^ | 98.21% (0.24)^3^ | 68.71% (5.06)^9^ | 0.7034 (0.0671) |
|  |  | 4 | 73.41% (4.40)^3^ | 97.81% (0.30)^3^ | 63.85% (3.30)^1^ | 0.6646 (0.0349) |
|  |  | 5 | 79.99% (4.19)^4^ | 97.44% (0.30)^3^ | 62.26% (3.58)^3^ | 0.6819 (0.0363) |
|  | COT | 0.5 | 27.52% (4.65)^1^ | 76.55% (3.55)^1^ | 37.45% (4.61)^7^ | 0.0435 (0.0529) |
|  |  | 1 | 30.10% (5.54)^1^ | 78.85% (3.52)^1^ | 41.94% (4.86)^7^ | 0.0959 (0.0554) |
|  |  | 2 | 44.08% (7.23)^1^ | 82.65% (3.46)^1^ | 56.29% (4.59)^7^ | 0.2814 (0.0618) |
|  |  | 3 | 63.56% (7.17)^1^ | 88.27% (3.65)^1^ | 73.69% (5.73)^1^ | 0.5353 (0.0668) |
|  |  | 4 | 76.50% (7.20)^1^ | 92.95% (2.51)^1^ | 84.76% (4.67)^1^ | 0.7104 (0.0640) |
|  |  | 5 | 79.48% (8.74)^1^ | 95.86% (2.14)^7^ | 90.94% (4.21)^1^ | 0.7755 (0.0708) |
|  | MMOT | 0.5 | 5.76% (1.56)^1^ | 93.99% (0.83)^1^ | 32.79% (8.92)^7^ | -0.0032 (0.0296) |
|  |  | 1 | 7.15% (1.80)^1^ | 94.68% (0.96)^1^ | 40.49% (9.84)^7^ | 0.0229 (0.0329) |
|  |  | 2 | 9.77% (1.79)^1^ | 96.86% (0.98)^1^ | 61.44% (10.18)^7^ | 0.0838 (0.0305) |
|  |  | 3 | 11.01% (3.13)^1^ | 98.82% (0.77)^1^ | 81.43% (13.11)^1^ | 0.1248 (0.0454) |
|  |  | 4 | 7.14% (1.86)^1^ | 98.11% (0.70)^1^ | 65.66% (10.52)^1^ | 0.0672 (0.0251) |
|  |  | 5 | 8.81% (2.13)^1^ | 96.26% (0.70)^7^ | 54.05% (9.08)^1^ | 0.0639 (0.0326) |
| **Type c** | sBIV | 0.5 | 0.82% (0.66)^3^ | 99.68% (0.02)^3^ | 11.46% (8.78)^10^ | 0.0089 (0.0120) |
|  |  | 1 | 1.61% (0.97)^6^ | 99.68% (0.02)^3^ | 20.05% (10.36)^2^ | 0.0228 (0.0172) |
|  |  | 2 | 7.53% (1.99)^6^ | 99.68% (0.02)^3^ | 54.42% (7.49)^6^ | 0.1216 (0.0318) |
|  |  | 3 | 20.40% (3.13)^3^ | 99.69% (0.03)^3^ | 76.95% (3.71)^3^ | 0.3075 (0.0420) |
|  |  | 4 | 35.61% (3.75)^3^ | 99.68% (0.02)^3^ | 85.30% (1.41)^1^ | 0.4865 (0.0398) |
|  |  | 5 | 52.06% (4.47)^3^ | 99.68% (0.02)^3^ | 89.39% (0.98)^3^ | 0.6441 (0.0384) |
|  | mBIV | 0.5 | 0.73% (0.63)^3^ | 99.72% (0.02)^3^ | 11.32% (9.26)^10^ | 0.0079 (0.0115) |
|  |  | 1 | 1.45% (0.90)^6^ | 99.72% (0.02)^3^ | 20.14% (10.71)^2^ | 0.0208 (0.0161) |
|  |  | 2 | 7.18% (1.95)^6^ | 99.73% (1.95)^3^ | 57.05% (0.03)^6^ | 0.1177 (0.0316) |
|  |  | 3 | 20.08% (3.11)^3^ | 99.72% (0.02)^3^ | 78.57% (3.73)^3^ | 0.3052 (0.0420) |
|  |  | 4 | 35.28% (3.71)^3^ | 99.71% (0.02)^3^ | 86.36% (1.46)^1^ | 0.4853 (0.0397) |
|  |  | 5 | 51.55% (4.38)^3^ | 99.71% (0.02)^3^ | 90.20% (0.94)^3^ | 0.6423 (0.0379) |
|  | SMOM | 0.5 | 10.18% (2.43)^3^ | 95.95% (0.11)^3^ | 11.64% (2.64)^10^ | 0.0651 (0.0262) |
|  |  | 1 | 20.83% (3.02)^6^ | 96.36% (0.12)^3^ | 23.10% (2.98)^2^ | 0.1803 (0.0311) |
|  |  | 2 | 44.89% (3.53)^6^ | 97.37% (0.16)^3^ | 47.25% (2.90)^6^ | 0.4326 (0.0326) |
|  |  | 3 | 62.28% (3.54)^3^ | 98.30% (0.15)^3^ | 65.81% (2.55)^3^ | 0.6212 (0.0288) |
|  |  | 4 | 71.58% (3.04)^3^ | 98.87% (0.13)^3^ | 76.98% (2.24)^1^ | 0.7285 (0.0241) |
|  |  | 5 | 79.22% (2.75)^3^ | 99.26% (0.13)^3^ | 84.93% (2.38)^3^ | 0.8103 (0.0226) |
|  | MMOM | 0.5 | 21.88% (2.44)^3^ | 96.48% (0.12)^3^ | 24.61% (2.63)^10^ | 0.1937 (0.0262) |
|  |  | 1 | 21.88% (3.09)^6^ | 96.49% (0.15)^3^ | 24.61% (3.02)^2^ | 0.1937 (0.0316) |
|  |  | 2 | 44.39% (3.16)^6^ | 97.27% (0.19)^3^ | 46.05% (2.74)^6^ | 0.4236 (0.0294) |
|  |  | 3 | 57.87% (3.65)^3^ | 97.84% (0.22)^3^ | 58.53% (3.23)^3^ | 0.5598 (0.0329) |
|  |  | 4 | 66.57% (3.50)^3^ | 98.18% (0.18)^3^ | 65.79% (2.95)^1^ | 0.6437 (0.0317) |
|  |  | 5 | 70.56% (3.87)^3^ | 98.30% (0.18)^3^ | 68.58% (3.12)^3^ | 0.6791 (0.0344) |
|  | COT | 0.5 | 29.41% (4.52)^1^ | 79.28% (3.03)^1^ | 42.00% (4.47)^7^ | 0.0936 (0.0525) |
|  |  | 1 | 29.41% (5.23)^1^ | 79.28% (3.90)^1^ | 42.00% (4.82)^7^ | 0.0936 (0.0519) |
|  |  | 2 | 37.44% (4.67)^1^ | 84.88% (3.47)^1^ | 56.04% (5.21)^1^ | 0.2428 (0.0500) |
|  |  | 3 | 46.87% (5.70)^1^ | 89.70% (2.83)^1^ | 70.19% (5.48)^1^ | 0.3973 (0.0558) |
|  |  | 4 | 56.03% (6.11)^1^ | 93.09% (2.64)^1^ | 80.84% (5.84)^1^ | 0.5302 (0.0578) |
|  |  | 5 | 63.00% (6.78)^1^ | 95.25% (2.28)^1^ | 87.38% (5.05)^1^ | 0.6242 (0.0614) |
|  | MMOT | 0.5 | 6.17% (1.54)^1^ | 94.45% (0.94)^1^ | 36.29% (9.36)^7^ | 0.0078 (0.0299) |
|  |  | 1 | 7.57% (1.68)^1^ | 95.29% (0.96)^1^ | 44.99% (9.34)^7^ | 0.0358 (0.0297) |
|  |  | 2 | 10.15% (2.12)^1^ | 97.28% (0.84)^1^ | 65.29% (10.37)^1^ | 0.0938 (0.0322) |
|  |  | 3 | 7.86% (2.38)^1^ | 97.66% (0.64)^1^ | 62.28% (10.55)^1^ | 0.0701 (0.0325) |
|  |  | 4 | 8.44% (2.07)^1^ | 97.60% (0.75)^1^ | 64.14% (9.26)^1^ | 0.0767 (0.0272) |
|  |  | 5 | 11.06% (2.29)^1^ | 97.14% (0.79)^1^ | 65.96% (9.12)^1^ | 0.1031 (0.0334) |
| **ALL** | sBIV | 0.5 | 0.77% (0.27)^3^ | 99.71% (0.04)^3^ | 31.79% (10.26)^5^ | 0.0081 (0.0050 |
|  |  | 1 | 1.14% (0.43)^3^ | 99.72% (0.04)^3^ | 40.64% (11.12)^6^ | 0.0143 (0.0074) |
|  |  | 2 | 4.11% (0.81)^3^ | 99.71% (0.04)^6^ | 71.03% (6.00)^6^ | 0.0623 (0.0132) |
|  |  | 3 | 13.12% (1.60)^3^ | 99.71% (0.04)^6^ | 88.84% (1.80)^8^ | 0.1975 (0.0225) |
|  |  | 4 | 32.98% (1.86)^1^ | 99.71% (0.04)^4^ | 95.25% (0.63)^9^ | 0.4471 (0.0209) |
|  |  | 5 | 67.18% (1.93)^5^ | 99.71% (0.04)^9^ | 97.65% (0.30)^3^ | 0.7674 (0.0151) |
|  | mBIV | 0.5 | 0.69% (0.24)^3^ | 99.74% (0.04)^3^ | 31.81% (10.39)^5^ | 0.0072 (0.0045) |
|  |  | 1 | 1.00% (0.38)^3^ | 99.75% (0.04)^3^ | 40.33% (11.74)^6^ | 0.0125 (0.0068) |
|  |  | 2 | 3.80% (0.79)^3^ | 99.76% (0.04)^6^ | 72.74% (6.27)^6^ | 0.0582 (0.0129) |
|  |  | 3 | 12.68% (1.52)^3^ | 99.75% (0.04)^6^ | 89.93% (1.86)^8^ | 0.1922 (0.0216) |
|  |  | 4 | 31.95% (1.86)^1^ | 99.75% (0.04)^4^ | 95.79% (0.72)^9^ | 0.4367 (0.0211) |
|  |  | 5 | 65.40% (2.02)^5^ | 99.75% (0.04)^9^ | 97.92% (0.34)^3^ | 0.7546 (0.0161) |
|  | SMOM | 0.5 | 8.17% (1.03)^3^ | 96.31% (0.16)^3^ | 28.07% (3.08)^5^ | 0.0632 (0.0156) |
|  |  | 1 | 13.87% (1.22)^3^ | 97.22% (0.16)^3^ | 46.76% (2.93)^6^ | 0.1560 (0.0172) |
|  |  | 2 | 28.97% (1.68)^3^ | 98.75% (0.13)^6^ | 80.41% (1.86)^6^ | 0.3762 (0.0194) |
|  |  | 3 | 43.53% (1.72)^3^ | 99.44% (0.08)^6^ | 93.16% (0.89)^8^ | 0.5502 (0.0171) |
|  |  | 4 | 54.25% (1.74)^1^ | 99.71% (0.06)^4^ | 97.03% (0.59)^9^ | 0.6591 (0.0157) |
|  |  | 5 | 62.48% (1.64)^5^ | 99.87% (0.04)^9^ | 98.82% (0.40)^3^ | 0.7346 (0.0135) |
|  | MMOM | 0.5 | 8.87% (1.08)^3^ | 96.45% (0.18)^3^ | 30.56% (3.31)^5^ | 0.0751 (0.0166) |
|  |  | 1 | 15.02% (1.48)^3^ | 97.29% (0.19)^3^ | 49.39% (3.71)^6^ | 0.1724 (0.0215) |
|  |  | 2 | 30.31% (1.67)^3^ | 98.77% (0.16)^6^ | 81.33% (2.49)^6^ | 0.3920 (0.0210) |
|  |  | 3 | 40.19% (2.92)^3^ | 99.43% (0.13)^6^ | 92.53% (1.70)^8^ | 0.5161 (0.0308) |
|  |  | 4 | 44.35% (2.43)^1^ | 99.64% (0.09)^4^ | 95.54% (1.23)^9^ | 0.5641 (0.0254) |
|  |  | 5 | 45.82% (2.61)^5^ | 99.73% (0.10)^9^ | 96.71% (1.20)^3^ | 0.5812 (0.0264) |
|  | COT | 0.5 | 25.87% (3.87)^1^ | 78.59% (4.82)^1^ | 76.50% (4.14)^1^ | 0.0285 (0.0296) |
|  |  | 1 | 25.86% (3.76)^1^ | 83.44% (5.08)^1^ | 80.98% (4.20)^7^ | 0.0592 (0.0296) |
|  |  | 2 | 30.55% (4.95)^1^ | 90.01% (3.99)^1^ | 89.31% (3.18)^1^ | 0.1333 (0.0334) |
|  |  | 3 | 34.12% (5.05)^1^ | 94.88% (2.87)^1^ | 94.82% (2.71)^7^ | 0.1893 (0.0402) |
|  |  | 4 | 34.85% (5.37)^1^ | 97.91% (1.92)^1^ | 97.91% (1.79)^7^ | 0.2129 (0.0419) |
|  |  | 5 | 34.22% (5.72)^1^ | 98.69% (1.43)^1^ | 98.62% (1.40)^1^ | 0.2154 (0.0468) |
|  | MMOT | 0.5 | 6.02% (0.78)^1^ | 95.41% (1.96)^1^ | 77.92% (9.53)^1^ | 0.0079 (0.0148) |
|  |  | 1 | 6.15% (0.99)^1^ | 96.61% (1.61)^1^ | 82.92% (8.17)^7^ | 0.0156 (0.0126) |
|  |  | 2 | 6.16% (1.16)^1^ | 99.41% (0.76)^1^ | 96.46% (4.75)^1^ | 0.0312 (0.0088) |
|  |  | 3 | 4.19% (1.76)^1^ | 99.65% (0.57)^1^ | 96.15% (6.91)^7^ | 0.0213 (0.0112) |
|  |  | 4 | 2.33% (0.73)^1^ | 99.82% (0.45)^1^ | 97.46% (6.24)^7^ | 0.0118 (0.0049) |
|  |  | 5 | 2.47% (0.79)^1^ | 99.99% (0.09)^1^ | 99.88% (1.25)^1^ | 0.0137 (0.0047 |
| *Abbreviations: TARGet Kids!, The Applied Research Group for Kids; SD, standard deviation; sBIV, static WHO cut-off values for biologically implausible values detection method; mBIV, modified method for biologically implausible values detection; SMOM, single-model outlier measurement detection method; MMOM, multi-model outlier measurement detection method; COT, clustering-based outlier trajectory detection method;* *MMOT, multi-model outlier trajectory detection method.*  *^1^ p-value < 0.2% for the overall association and all possible combinations (mmom-mbiv, sbiv-mbiv, smom-mbiv, sBIV-mmom, smom-mmom, smom-sbiv) or < 0.2% for the comparison between cot and mmot*  *^2^ p-value < 0.2% for the overall association and for the combinations sbiv-mbiv, smom-mbiv only*  *^3^ p-value < 0.2% for the overall association and but not for combinations sbiv-mbiv*  *^4^p-value < 0.2% for the overall association and but not for combination sbiv-smom*  *^5^ p-value < 0.2% for the overall association and but not for all combinations*  *^6^ p-value < 0.2% for the overall association and but not for combinations sbiv-mbiv and smom-mmom*  *^7^p-value > 0.2% for the comparison between cot and mmot*  *^8^p-value < 0.2% for the overall association and but not for combination mmom-smom*  *^9^p-value < 0.2% for the overall association and but not for combination mmom-sbiv, mmom-mbiv*  *^10^ p-value < 0.2% for the overall association and all possible combinations (mmom-mbiv, sbiv-mbiv, smom-mbiv, sBIV-mmom, smom-mmom, smom-sbiv)* | | | | | | |

**Supplemental Table 3a:** Summary of results from 100 simulation experiments of outlier detection methods applied on the CTX Dataset. Expressed as mean (SD) for MUAC z-scores.

| **Outlier type** | **Detection method** | **SD error** | **Sensitivity** | **Specificity** | **Precision** | **Kappa** |
| --- | --- | --- | --- | --- | --- | --- |
|  |  |  |  |  |  |  |
| **Type a** | sBIV | 0.5 | 0.29%^3^ (0.29) | 99.86%^5^ (0.01) | 9.68%^6^ (9.04) | 0.0029 (0.0055) |
|  |  | 1 | 0.58%^3^ (0.40) | 99.86%^5^ (0.01) | 16.95%^11^ (10.17) | 0.0081 (0.0073) |
|  |  | 2 | 2.45%^3^ (0.81) | 99.86%^5^ (0.01) | 46.89%^4^ (8.89) | 0.0419 (0.0144) |
|  |  | 3 | 8.44%^3^ (1.64) | 99.86%^5^  (0.01) | 75.47%^5^ (4.04) | 0.1430 (0.0264) |
|  |  | 4 | 20.58%^3^ (2.18) | 99.86%^5^ (0.01) | 88.65%^5^ (1.41) | 0.3209 (0.0291) |
|  |  | 5 | 39.34%^3^ (2.57) | 99.86%^5^ (0.01) | 93.72%^5^ (0.62) | 0.5402 (0.0263) |
|  | mBIV | 0.5 | 0.08%^3^ (0.13) | 99.99%^5^ (0.01) | NA | 0.0012 (0.0025) |
|  |  | 1 | 0.22%^3^ (0.28) | 99.98%^5^ (0.01) | 32.21%^11^ (31.03) | 0.0038 (0.0052) |
|  |  | 2 | 2.11%^3^ (0.76) | 99.98%^5^ (0.01) | 86.19%^4^ (7.17) | 0.0389 (0.0138) |
|  |  | 3 | 8.34%^3^ (1.62) | 99.98%^5^ (0.01) | 95.71%^5^ (2.49) | 0.1463 (0.0265) |
|  |  | 4 | 20.45%^3^ (2.15) | 99.98%^5^ (0.01) | 97.98%^5^ (1.02) | 0.3263 (0.0291) |
|  |  | 5 | 39.11%^3^ (2.58) | 99.98%^5^ (0.01) | 98.94%^5^ (0.56) | 0.5473 (0.0267) |
|  | SMOM | 0.5 | 6.72%^3^ (1.30) | 94.95%^5^ (0.07) | 6.54%^6^ (1.24) | 0.0165 (0.0132) |
|  |  | 1 | 11.28%^3^ (1.55) | 95.25%^5^ (0.07) | 11.10%^11^ (1.45) | 0.0647 (0.0156) |
|  |  | 2 | 32.42%^3^ (2.26) | 96.31%^5^ (0.09) | 31.59%^4^ (1.83) | 0.2837 (0.0211) |
|  |  | 3 | 60.06%^3^ (2.18) | 97.34%^5^ (0.09) | 54.28%^5^ (1.44) | 0.5464 (0.0179) |
|  |  | 4 | 82.27%^3^ (1.81) | 98.17%^5^ (0.09) | 70.28%^5^ (1.25) | 0.7442 (0.0144) |
|  |  | 5 | 93.46%^3^ (1.08) | 98.83%^3^ (0.06) | 80.79%^5^ (0.86) | 0.8591 (0.0083) |
|  | MMOM | 0.5 | 7.66%^3^ (1.08) | 95.28%^5^ (0.10) | 7.86%^6^ (1.07) | 0.0297 (0.0111) |
|  |  | 1 | 15.40%^3^ (2.07) | 95.62%^5^ (0.11) | 15.59%^11^ (1.90) | 0.1107 (0.0206) |
|  |  | 2 | 45.63%^3^ (2.52) | 96.69%^5^ (0.10) | 42.07%^4^ (1.72) | 0.4069 (0.0214) |
|  |  | 3 | 73.65%^3^ (2.63) | 97.96%^5^ (0.11) | 65.50%^5^ (1.54) | 0.6761 (0.0192) |
|  |  | 4 | 73.22%^3^ (6.55) | 98.60%^5^ (0.14) | 73.20%^5^ (2.98) | 0.7174 (0.0474) |
|  |  | 5 | 73.79%^3^ (2.45) | 98.17%^5^ (0.22) | 68.09%^5^ (2.86) | 0.6920 (0.0247) |
|  | COT | 0.5 | 27.66%^1^ (2.72) | 75.51%^1^ (2.79) | 39.95%^7^ (2.95) | 0.0340 (0.0319) |
|  |  | 1 | 30.79%^1^ (3.46) | 77.69%^1^ (2.73) | 44.61%^1^ (3.48) | 0.0906 (0.0390) |
|  |  | 2 | 51.43%^1^ (4.51) | 85.41%^1^ (2.49) | 67.60%^1^ (3.56) | 0.3871 (0.0427) |
|  |  | 3 | 71.90%^1^ (5.40) | 91.09%^1^ (1.77) | 82.71%^1^ (2.73) | 0.6467 (0.0464) |
|  |  | 4 | 79.38%^1^ (5.28) | 94.55%^1^ (1.58) | 89.62%^1^ (2.68) | 0.7565 (0.0480) |
|  |  | 5 | 76.46%^1^ (5.12) | 96.85%^1^ (1.45) | 93.55%^1^ (2.83) | 0.7610 (0.0490) |
|  | MMOT | 0.5 | 3.87%^1^ (0.82) | 96.52%^1^ (0.52) | 39.58%^7^ (8.26) | 0.0049 (0.0160) |
|  |  | 1 | 4.21%^1^ (0.85) | 96.57%^1^ (0.52) | 41.70%^1^ (7.74) | 0.0097 (0.0150) |
|  |  | 2 | 5.56%^1^ (1.05) | 96.87%^1^ (0.55) | 51.01%^1^ (8.17) | 0.0298 (0.0177) |
|  |  | 3 | 7.49%^1^ (1.21) | 97.90%^1^ (0.43) | 67.68%^1^ (5.82) | 0.0659 (0.0158) |
|  |  | 4 | 9.08%^1^ (2.04) | 98.25%^1^ (0.48) | 74.57%^1^ (8.81) | 0.0893 (0.0280) |
|  |  | 5 | 5.81%^1^ (1.33) | 97.10%^1^ (0.83) | 54.45%^1^ (8.49) | 0.0356 (0.0178) |
| **Type b** | sBIV | 0.5 | 0.47%^13^ (0.35) | 99.86%^5^ (0.01) | 14.15%^11^ (9.64) | 0.0060 (0.0065) |
|  |  | 1 | 1.16%^1^ (0.51) | 99.86%^5^ (0.09) | 29.50%^8^ (9.71) | 0.0187 (0.0093) |
|  |  | 2 | 4.93%^3^ (1.01) | 99.86%^5^ (0.01) | 64.66%^5^ (5.34) | 0.0850 (0.0174) |
|  |  | 3 | 16.91%^3^ (2.00) | 99.86%^3^ (0.01) | 86.51%^5^ (1.67) | 0.2706 (0.0281) |
|  |  | 4 | 40.97%^3^ (2.50) | 99.86%^3^ (0.01) | 93.97%^5^ (0.56) | 0.5568 (0.0252) |
|  |  | 5 | 79.05%^3^ (1.79) | 99.86%^5^ (0.01) | 96.78%^5^ (0.24) | 0.8640 (0.0116) |
|  | mBIV | 0.5 | 0.15%^13^ (0.20) | 99.99%^5^ (0.003) | 25.13%^11^ (30.78) | 0.0026 (0.0038) |
|  |  | 1 | 0.39%^1^ (0.30) | 99.99%^5^ (0.004) | 52.02%^8^ (30.37) | 0.0072 (0.0057) |
|  |  | 2 | 4.15%^3^ (1.05) | 99.99%^5^ (0.005) | 93.42%^5^ (2.35) | 0.0753 (0.0184) |
|  |  | 3 | 16.57%^3^ (1.97) | 99.98%^3^  (0.01) | 97.83%^5^ (1.16) | 0.2724 (0.0282) |
|  |  | 4 | 40.32%^3^ (2.43) | 99.98%^3^ (0.01) | 99.00%^5^ (0.48) | 0.5600 (0.0251) |
|  |  | 5 | 78.11%^3^ (1.80) | 99.98%^5^ (0.01) | 99.44%^5^ (0.21) | 0.8690 (0.0118) |
|  | SMOM | 0.5 | 9.67%^13^ (1.42) | 95.16%^5^ (0.07) | 9.52%^11^ (1.34) | 0.0479 (0.0144) |
|  |  | 1 | 17.14%^1^ (2.04) | 95.54%^5^ (0.10) | 16.83%^8^ (1.91) | 0.1257 (0.0206) |
|  |  | 2 | 37.37%^3^ (2.33) | 96.62%^5^ (0.10) | 36.80%^5^ (1.85) | 0.3374 (0.0214) |
|  |  | 3 | 63.76%^3^ (2.10) | 97.58%^3^ (0.08) | 58.06%^5^ (1.39) | 0.5860 (0.0172) |
|  |  | 4 | 88.46%^3^ (1.59) | 98.37%^3^ (0.08) | 74.06%^5^ (1.00) | 0.7950 (0.0108) |
|  |  | 5 | 98.26%^3^ (0.70) | 99.02%^5^ (0.06) | 84.06%^5^ (0.81) | 0.9007 (0.0063) |
|  | MMOM | 0.5 | 10.16%^13^ (0.20) | 95.35%^5^ (0.10) | 10.32%^11^ (1.36) | 0.0556 (0.0142) |
|  |  | 1 | 19.34%^1^ (1.84) | 95.78%^5^ (0.11) | 19.42%^8^ (1.70) | 0.1514 (0.0183) |
|  |  | 2 | 46.96%^3^ (2.49) | 96.82%^5^ (0.10) | 43.72%^5^ (1.69) | 0.4229 (0.0210) |
|  |  | 3 | 70.88%^3^ (3.70) | 97.95%^3^ (0.13) | 64.57%^5^ (2.41) | 0.6577 (0.0304) |
|  |  | 4 | 66.14%^3^ (5.56) | 98.36%^3^ (0.14) | 67.95%^5^ (2.90) | 0.6527 (0.0420) |
|  |  | 5 | 68.96%^3^ (3.01) | 98.44%^5^ (0.19) | 70.04%^5^ (2.77) | 0.6787 (0.0256) |
|  | COT | 0.5 | 27.39%^1^ (2.81) | 75.82%^1^ (2.98) | 40.02%^1^ (2.96) | 0.0344 (0.0334) |
|  |  | 1 | 32.36%^1^ (3.62) | 78.32%^1^ (3.11) | 46.87%^1^ (3.42) | 0.1140 (0.0385) |
|  |  | 2 | 52.41%^1^ (4.80) | 85.44%^1^ (2.30) | 67.79%^1^ (3.70) | 0.3969 (0.0487) |
|  |  | 3 | 63.16%^1^ (6.53) | 91.98%^1^ (2.01) | 82.26%^1^ (3.91) | 0.5783 (0.0651) |
|  |  | 4 | 62.87%^1^ (6.314) | 94.71%^1^ (1.79) | 87.50%^1^ (3.90) | 0.6106 (0.0647) |
|  |  | 5 | 56.78%^1^ (5.73) | 96.01%^1^ (1.41) | 89.34%^1^ (3.53) | 0.5701 (0.0598) |
|  | MMOT | 0.5 | 3.98%^1^ (1.01) | 96.50%^1^ (0.57) | 39.94%^1^ (9.70) | 0.0059 (0.0188) |
|  |  | 1 | 4.57%^1^ (1.03) | 96.64%^1^ (0.60) | 44.43%^1^ (9.51) | 0.0149 (0.0193) |
|  |  | 2 | 6.03%^1^ (1.03) | 97.05%^1^ (0.44) | 54.22%^1^ (6.97) | 0.0379 (0.0160) |
|  |  | 3 | 7.22%^1^ (1.09) | 91.98%^1^ (0.45) | 82.26%^1^ (6.73) | 0.5783 (0.0152) |
|  |  | 4 | 8.33%^1^ (1.32) | 98.01%^1^ (0.42) | 70.81%^1^ (6.44) | 0.0775 (0.0181) |
|  |  | 5 | 6.64%^1^ (1.74) | 98.26%^1^ (0.76) | 69.94%^1^ (8.95) | 0.0602 (0.0187) |
| **Type c** | sBIV | 0.5 | 0.62%^14^ (0.40) | 99.86%^5^ (0.01) | 18.15%^11^ (10.06) | 0.0089 (0.0074) |
|  |  | 1 | 1.65%^1^ (0.56) | 99.86%^5^ (0.01) | 37.59%^8^ (9.12) | 0.0275 (0.0103) |
|  |  | 2 | 5.26%^3^ (1.21) | 99.86%^5^ (0.01) | 65.84%^5^ (5.94) | 0.0907 (0.0207) |
|  |  | 3 | 14.41%^3^ (1.79) | 99.86%^15^ (0.01) | 84.19%^1^ (1.96) | 0.2346 (0.0263) |
|  |  | 4 | 27.55%^16^ (2.30) | 99.86%^5^ (0.01) | 91.29%^5^ (0.86) | 0.4092 (0.0277) |
|  |  | 5 | 42.59%^3^ (2.35) | 99.86%^5^ (0.01) | 94.21%^5^ (0.50) | 0.5730 (0.0233) |
|  | mBIV | 0.5 | 0.28%^14^ (0.28) | 99.99%^5^ (0.004) | NA | 0.0051 (0.0053) |
|  |  | 1 | 0.97%^1^ (0.43) | 99.99%^5^ (0.002) | 75.18%^8^ (12.70) | 0.0180 (0.0079) |
|  |  | 2 | 4.70%^3^ (1.15) | 99.99%^5^ (0.01) | 94.10%^5^ (2.98) | 0.0850 (0.0201) |
|  |  | 3 | 13.82%^3^ (1.73) | 99.98%^15^ (0.01) | 97.26%^1^ (1.18) | 0.2321 (0.0258) |
|  |  | 4 | 26.83%^16^ (2.30) | 99.98%^5^ (0.01) | 98.49%^5^ (0.86) | 0.4086 (0.0283) |
|  |  | 5 | 41.75%^3^ (2.26) | 99.98%^5^ (0.01) | 99.05%^5^ (0.52) | 0.5744 (0.0228) |
|  | SMOM | 0.5 | 9.45%^14^ (1.58) | 95.12%^5^ (0.08) | 9.25%^11^ (1.51) | 0.0452 (0.0162) |
|  |  | 1 | 15.73%^1^ (1.60) | 95.45%^5^ (0.08) | 15.38%^8^ (1.47) | 0.1106 (0.0160) |
|  |  | 2 | 29.61%^3^ (2.31) | 96.44%^5^ (0.12) | 30.44%^5^ (2.20) | 0.2639 (0.0234) |
|  |  | 3 | 44.58% ^3^ (2.28) | 97.33%^15^ (0.12) | 46.79%^1^ (2.02) | 0.4286 (0.0217) |
|  |  | 4 | 57.05%^16^ (2.11) | 98.08%^5^ (0.14) | 61.01%^5^ (2.09) | 0.5687 (0.0199) |
|  |  | 5 | 68.35%^3^ (2.06) | 98.74%^5^ (0.09) | 74.01%^5^ (1.50) | 0.6959 (0.0154) |
|  | MMOM | 0.5 | 9.74%^14^ (1.36) | 95.34%^5^ 0.09) | 9.91%^11^ (1.35) | 0.0512 (0.0142) |
|  |  | 1 | 16.40%^1^ (1.59) | 95.73%^5^ (0.11) | 16.80%^8^ (1.53) | 0.1226 (0.0162) |
|  |  | 2 | 33.98%^3^ (2.34) | 96.60%^5^ (0.15) | 34.45%^5^ (2.07) | 0.3077 (0.0223) |
|  |  | 3 | 48.07%^3^ (2.54) | 97.25%^15^ (0.17) | 47.95%^1^ (2.38) | 0.4526 (0.0245) |
|  |  | 4 | 56.03%^16^ (2.18) | 97.96%^5^ (0.18) | 59.16%^5^ (2.57) | 0.5536 (0.0225) |
|  |  | 5 | 62.61%^3^ (2.74) | 98.32%^5^ (0.13) | 66.29%^5^ (2.21) | 0.6256 (0.0240) |
|  | COT | 0.5 | 27.08%^1^ (2.88) | 75.54%^1^ (2.74) | 39.39%^1^ (2.69) | 0.0281 (0.0308) |
|  |  | 1 | 30.13%^1^ (3.00) | 78.60%^1^ (2.73) | 45.39%^1^ (3.32) | 0.0937 (0.0353) |
|  |  | 2 | 40.12%^1^ (3.59) | 85.81%^1^ (2.38) | 62.60%^1^ (3.76) | 0.2798 (0.0378) |
|  |  | 3 | 49.68%^1^ (5.20) | 91.15%^1^ (1.84) | 76.60%^1^ (4.04) | 0.4401 (0.0545) |
|  |  | 4 | 54.67%^1^ (4.24) | 93.95%^1^ (1.52) | 84.20%^1^ (3.42) | 0.5237 (0.0455) |
|  |  | 5 | 57.43%^1^ (5.63) | 95.66%^1^ (1.46) | 88.68%^1^ (3.38) | 0.5720 (0.0548) |
|  | MMOT | 0.5 | 4.10%^1^ (0.88) | 96.55%^1^ (0.54) | 40.98%^1^ (8.42) | 0.0079 (0.0163) |
|  |  | 1 | 4.83%^1^ (0.91) | 96.77%^1^ (0.52) | 46.75%^1^ (8.06) | 0.0196 (0.0164) |
|  |  | 2 | 6.24%^1^ (1.16) | 97.45%^1^ (0.66) | 59.06%^1^ (8.36) | 0.0451 (0.0176) |
|  |  | 3 | 8.06%^1^ (1.22) | 97.37%^1^ (0.66) | 64.22%^1^ (7.19) | 0.0663 (0.0174) |
|  |  | 4 | 7.38%^1^ (1.20) | 97.05%^1^ (0.52) | 59.39%^1^ (6.49) | 0.0540 (0.0173) |
|  |  | 5 | 7.99%^1^ (1.20) | 97.06%^1^ (0.69) | 61.56%^1^ (7.28) | 0.0615 (0.0183) |
| **ALL** | sBIV | 0.5 | 0.45%^6^ (0.20) | 99.86%^1^ (0.02) | 35.00%^8^ (12.95) | 0.0052 (0.0035) |
|  |  | 1 | 0.55%^1^ (0.30) | 99.99%^1^ (0.02) | 85.77%^8^ (7.73) | 0.0090 (0.0051) |
|  |  | 2 | 4.39%^5^ (0.64) | 99.86%^5^ (0.02) | 84.66%^5^ (2.74) | 0.0697 (0.0102) |
|  |  | 3 | 13.31%^5^ (1.02) | 99.86%^5^ (0.02) | 94.41%^5^ (0.82) | 0.2038 (0.0145) |
|  |  | 4 | 29.86%^10^ (1.31) | 99.86%^8^ (0.02) | 97.39%^11^ (0.36) | 0.4158 (0.0153) |
|  |  | 5 | 53.55%^5^ (1.17) | 99.86%^12^ (0.02) | 98.58%^1^ (0.20) | 0.6578 (0.0100) |
|  | mBIV | 0.5 | 0.15%^6^ (0.11) | 99.99%^1^ (0.01) | NA | 0.0024 (0.0020) |
|  |  | 1 | 1.00%^1^ (0.22) | 99.75%^1^ (0.01) | 40.33%^8^ (10.29) | 0.0125 (0.0038) |
|  |  | 2 | 3.65%^5^ (0.55) | 99.98%^5^ (0.01) | 96.78%^5^ (2.06) | 0.0600 (0.0089) |
|  |  | 3 | 12.58%^5^ (1.00) | 99.97%^5^ (0.02) | 98.52%^5^ (0.79) | 0.1957 (0.0144) |
|  |  | 4 | 28.63%^10^ (1.29) | 99.96%^8^ (0.02) | 99.29%^11^ (0.35) | 0.4044 (0.0153) |
|  |  | 5 | 51.81%^5^ (1.31) | 99.96%^12^ (0.02) | 99.58%^1^ (0.20) | 0.6450 (0.0120) |
|  | SMOM | 0.5 | 8.05%^6^ (0.86) | 95.41%^1^ (0.12) | 23.64%^8^ (2.30) | 0.0476 (0.0130) |
|  |  | 1 | 12.87%^1^ (0.91) | 96.35%^1^ (0.11) | 38.32%^8^ (2.18) | 0.1268 (0.0131) |
|  |  | 2 | 23.98%^5^ (1.01) | 97.99%^5^ (0.10) | 67.74%^5^ (1.72) | 0.2992 (0.0133) |
|  |  | 3 | 36.50%^5^ (1.01) | 99.16%^5^ (0.07) | 88.50%^5^ (0.98) | 0.4704 (0.0113) |
|  |  | 4 | 48.18%^10^ (1.08) | 99.76%^8^ (0.04) | 97.24%^11^ (0.40) | 0.6050 (0.0104) |
|  |  | 5 | 58.17%^5^ (1.17) | 99.94%^12^ (0.02) | 99.40%^1^ (0.20) | 0.7007 (0.0100) |
|  | MMOM | 0.5 | 8.33%^6^ (0.82) | 95.64%^1^ (0.14) | 25.19% ^8^ (2.26) | 0.0547 (0.0125) |
|  |  | 1 | 13.85%^1^ (0.88) | 96.51%^1^ (0.15) | 41.19%^8^ (2.18) | 0.1426 (0.0128) |
|  |  | 2 | 28.34%^5^ (1.16) | 98.42%^5^  (0.14) | 75.96%^5^ (1.86) | 0.3606 (0.0141) |
|  |  | 3 | 40.27%^5^ (1.69) | 99.35%^5^ (0.17) | 91.64%^5^ (2.04) | 0.5149 (0.0182) |
|  |  | 4 | 46.59%^10^ (2.22) | 99.77%^8^ (0.07) | 97.33% (0.81) | 0.5902 (0.0215) |
|  |  | 5 | 48.78%^5^ (1.63) | 99.89%^12^ (0.05) | 98.76%^1^ (0.58) | 0.6147 (0.0161) |
|  | COT | 0.5 | 26.08%^1^ (2.63) | 77.65%^1^ (3.68) | 79.38% ^7^ (2.84) | 0.0210 (0.0208) |
|  |  | 1 | 28.20%^1^ (2.98) | 82.93%^1^ (3.21) | 84.63%^1^  (2.63) | 0.0629 (0.0226) |
|  |  | 2 | 31.75%^1^ (3.36) | 91.65%^1^ (2.56) | 92.78%^1^ (2.02) | 0.1323 (0.0250) |
|  |  | 3 | 34.32%^1^ (3.77) | 96.07%^1^ (2.04) | 96.69%^1^ (1.67) | 0.1737 (0.0281) |
|  |  | 4 | 35.82%^1^ (3.33) | 97.99%^1^ (1.55) | 98.35%^1^ (1.25) | 0.1952 (0.0277) |
|  |  | 5 | 35.73%^1^ (3.60) | 98.93%^1^ (1.09) | 99.12%^1^ (0.88) | 0.1991 (0.0291) |
|  | MMOT | 0.5 | 3.88%^1^ (0.40) | 96.51%^1^ (1.10) | 78.57%^7^ (6.68) | 0.0019 (0.0068) |
|  |  | 1 | 4.31%^1^ (0.44) | 96.88%^1^ (1.19) | 82.20%^1^ (6.66) | 0.0056 (0.0069) |
|  |  | 2 | 4.79%^1^ (0.72) | 98.20%^1^ (0.82) | 89.90%^1^ (4.60) | 0.0141 (0.0054) |
|  |  | 3 | 4.79%^1^ (0.97) | 98.77%^1^ (0.82) | 92.78%^1^ (5.21) | 0.0168 (0.0061) |
|  |  | 4 | 2.43%^1^ (0.64) | 99.42% ^1^ (0.45) | 93.03%^1^ (5.57) | 0.0087 (0.0040) |
|  |  | 5 | 2.55%^1^ (0.55) | 99.73%^1^ (0.38) | 97.19%^1^ (3.95) | 0.0107 (0.0029) |
| *Abbreviations: CTX, the co-trimoxazole prophylaxis trial; SD, standard deviation; MUAC, mid-upper arm circumference-for-age z-scores; NA, not available; sBIV, static WHO cut-off values for biologically implausible values detection method; mBIV, modified method for biologically implausible values detection; SMOM, single-model outlier measurement detection method; MMOM, multi-model outlier measurement detection method; COT, clustering-based outlier trajectory detection method;* *MMOT, multi-model outlier trajectory detection method.*  *^1^ p-value < 0.2% for the overall association and all possible combinations (mmom-mbiv, sbiv-mbiv, smom-mbiv, sBIV-mmom, smom-mmom, smom-sbiv) or < 0.2% for the comparison between cot and mmot*  *^2^ p-value < 0.2% for the overall association and for the combinations sbiv-mbiv, smom-mbiv only*  *^3^ p-value < 0.2% for the overall association and but not for combinations sbiv-mbiv*  *^4^p-value < 0.2% for the overall association and for combination sbiv-mmom only*  *^5^ p-value < 0.2% for the overall association and but not for all combinations*  *^6^ p-value < 0.2% for the overall association and but not for combinations mmom-mbiv and smom-mbiv*  *^7^p-value > 0.2% for the comparison between cot and mmot*  *^8^p-value < 0.2% for the overall association and but not for combination mmom-smom*  *^9^p-value < 0.2% for the overall association and but not for combination mmom-sbiv, mmom-mbiv*  *^10^ p-value < 0.2% for the overall association and for combination sbiv-mbiv only*  *^11^ p-value < 0.2% for the overall association and for the combinations sbiv-mbiv, smom-mbiv, mmom-mbiv only*  *^12^ p-value < 0.2% for the overall association and for combination smom-mbiv only*  *^13^ p-value < 0.2% for the overall association but not for sbiv-mbiv, smom-sbiv*  *^14^ p-value < 0.2% for the overall association but not for sbiv-mbiv, smom-sbiv*  *^15^ p-value < 0.2% for the overall association and for combination mmom-smom only*  *^16^ p-value < 0.2% for the overall association but not for sbiv-mbiv, smom-mmom* | | | | | | |

**Supplemental Table 3b:** Summary of results from 100 simulation experiments of outlier detection methods applied on the CTX Dataset. Expressed as mean (SD) for weight-for age z-scores.

| **Outlier type** | **Detection method** | **SD error** | **Sensitivity** | **Specificity** | **Precision** | **Kappa** |
| --- | --- | --- | --- | --- | --- | --- |
|  |  |  |  |  |  |  |
| **Type a** | sBIV | 0.5 | 1.46% (0.90)^6^ | 98.81% (0.02)^6^ | 2.46% (1.50)^4^ | 0.0033 (0.0113) |
|  |  | 1 | 2.66% (1.42)^6^ | 98.81% (0.02)^1^ | 4.39% (2.30)^2^ | 0.0182 (0.0177) |
|  |  | 2 | 7.86% (2.27)^1^ | 98.81% (0.02)^6^ | 11.95% (3.12)^5^ | 0.0800 (0.0264) |
|  |  | 3 | 21.06% (3.12)^3^ | 98.81% (0.02)^1^ | 26.65% (3.04)^5^ | 0.2213 (0.0313) |
|  |  | 4 | 35.39% (4.28)^3^ | 98.80% (0.02)^1^ | 37.67% (2.94)^1^ | 0.3521 (0.0366) |
|  |  | 5 | 46.68% (4.62)^3^ | 98.81% (0.02)^6^ | 44.58% (2.69)^5^ | 0.4444 (0.0362) |
|  | mBIV | 0.5 | 0.06% (0.20)^6^ | 99.97% (0.004)^6^ | 3.50% (10.67)^4^ | 0.0007 (0.0038) |
|  |  | 1 | 0.36% (0.43)^6^ | 99.97% (0.005)^1^ | 16.22% (18.74)^2^ | 0.0063 (0.0083) |
|  |  | 2 | 6.41% (1.94)^1^ | 99.96% (0.02)^6^ | 74.85% (8.77)^5^ | 0.1148 (0.0330) |
|  |  | 3 | 20.85% (3.15)^3^ | 99.93% (0.03)^1^ | 86.90% (5.05)^5^ | 0.3300 (0.0042) |
|  |  | 4 | 35.22% (4.22)^3^ | 99.94% (0.02)^1^ | 92.64% (2.94)^1^ | 0.5037 (0.0462) |
|  |  | 5 | 46.39% (4.63)^3^ | 99.94% (0.03)^6^ | 94.09% (2.71)^5^ | 0.6151 (0.0441) |
|  | SMOM | 0.5 | 1.46% (1.68)^6^ | 98.81% (0.04)^6^ | 2.46% (0.76)^4^ | 0.0033 (0.0105) |
|  |  | 1 | 11.44% (2.74)^6^ | 95.48% (0.06)^1^ | 4.96% (1.21)^2^ | 0.0422 (0.0170) |
|  |  | 2 | 31.79% (3.71)^1^ | 95.87% (0.07)^6^ | 13.76% (1.57)^5^ | 0.1684 (0.0223) |
|  |  | 3 | 60.36% (3.09)^3^ | 96.42% (0.10)^1^ | 25.81% (1.54)^5^ | 0.3429 (0.0202) |
|  |  | 4 | 83.78% (2.60)^3^ | 97.04% (0.08)^1^ | 36.77% (1.41)^1^ | 0.4970 (0.072) |
|  |  | 5 | 95.45% (1.90)^3^ | 97.57% (0.08)^6^ | 44.81% (1.49)^5^ | 0.5987 (0.0162) |
|  | MMOM | 0.5 | 7.30% (2.00)^6^ | 95.24% (0.11)^6^ | 3.07% (0.87)^4^ | 0.0152 (0.01) |
|  |  | 1 | 16.46% (2.79)^6^ | 95.42% (0.11)^1^ | 6.90% (1.17)^2^ | 0.0708 (0.02) |
|  |  | 2 | 53.78% (3.35)^1^ | 95.93% (0.08)^6^ | 21.51% (1.41)^5^ | 0.2865 (0.0196) |
|  |  | 3 | 85.08% (3.14)^3^ | 96.57% (0.08)^1^ | 33.82% (1.33)^5^ | 0.4686 (0.0174) |
|  |  | 4 | 97.49% (1.45)^3^ | 97.23% (0.11)^1^ | 41.96% (1.48)^1^ | 0.5746 (0.0154) |
|  |  | 5 | 99.08% (1.20)^3^ | 98.00% (0.13)^6^ | 50.65% (2.10)^5^ | 0.6610 (0.0192) |
|  | COT | 0.5 | 28.12% (4.47)^1^ | 74.41% (2.51)^1^ | 16.20% (2.03)^4^ | 0.0194 (0.0318) |
|  |  | 1 | 37.11% (4.19)^1^ | 76.35% (2.48)^1^ | 21.71% (2.40)^1^ | 0.1044 (0.0354) |
|  |  | 2 | 72.27% (4.58)^1^ | 85.48% (2.46)^1^ | 47.02% (4.18)^1^ | 0.4724 (0.0441) |
|  |  | 3 | 92.87% (2.67)^1^ | 92.39% (1.61)^1^ | 68.52% (4.56)^1^ | 0.7432 (0.0403) |
|  |  | 4 | 98.81% (1.09)^1^ | 95.64% (1.52)^1^ | 80.34% (5.62)^1^ | 0.8622 (0.0423) |
|  |  | 5 | 99.83% (0.49)^1^ | 97.00% (1.28)^1^ | 85.73% (5.32)^1^ | 0.9063 (0.0372) |
|  | MMOT | 0.5 | 3.87% (1.41)^1^ | 96.30% (0.34)^1^ | 15.56% (5.73)^4^ | 0.0023 (0.0243) |
|  |  | 1 | 3.99% (1.74)^1^ | 96.28% (0.36)^1^ | 15.87% (6.79)^1^ | 0.0039 (0.0291) |
|  |  | 2 | 5.49% (1.87)^1^ | 96.59% (0.36)^1^ | 21.99% (7.05)^1^ | 0.0301 (0.0303) |
|  |  | 3 | 10.05% (2.11)^1^ | 97.03% (0.25)^1^ | 37.07% (6.13)^1^ | 0.1010 (0.0312) |
|  |  | 4 | 18.90% (2.19)^1^ | 97.50% (0.30)^1^ | 57.02% (4.58)^1^ | 0.2261 (0.0295) |
|  |  | 5 | 24.95% (3.42)^1^ | 98.46% (0.28)^1^ | 73.94% (4.11)^1^ | 0.3213 (0.0404) |
| **Type b** | sBIV | 0.5 | 2.48% (1.34)^2^ | 98.81% (0.02)^6^ | 4.08% (2.16)^3^ | 0.0159 (0.0167) |
|  |  | 1 | 4.78% (1.81)^6^ | 98.81% (0.02)^2^ | 7.58% (2.70)^3^ | 0.0437 (0.0218) |
|  |  | 2 | 15.56% (3.01)^6^ | 98.81% (0.02)^6^ | 21.09% (3.45)^4^ | 0.1647 (0.0325) |
|  |  | 3 | 41.65% (4.79)^5^ | 98.81% (0.02)^2^ | 41.97% (3.17)^1^ | 0.4058 (0.0398) |
|  |  | 4 | 70.25% (3.50)^5^ | 98.81% (0.02)^1^ | 54.85% (1.58)^1^ | 0.6070 (0.0227) |
|  |  | 5 | 92.60% (2.21)^5^ | 98.80% (0.02)^2^ | 61.39% (1.09)^6^ | 0.7318 (0.0135) |
|  | mBIV | 0.5 | 0.19% (0.34)^2^ | 99.97% (0.004)^6^ | 10.17% (18.49)^3^ | 0.0031 (0.0066) |
|  |  | 1 | 0.58% (0.58)^6^ | 99.97% (0.002)^2^ | 25.18% (22.14)^3^ | 0.0106 (0.0111) |
|  |  | 2 | 12.14% (2.49)^6^ | 99.96% (0.01)^6^ | 86.78% (4.26)^4^ | 0.2084 (0.0382) |
|  |  | 3 | 40.83% (4.72)^5^ | 99.94% (0.03)^2^ | 93.58% (2.78)^1^ | 0.5616 (0.0472) |
|  |  | 4 | 69.46% (3.47)^5^ | 99.94% (0.03)^1^ | 95.94% (1.89)^1^ | 0.8019 (0.0251) |
|  |  | 5 | 91.52% (2.37)^5^ | 99.94% (0.02)^2^ | 97.22% (1.13)^6^ | 0.9415 (0.0138) |
|  | SMOM | 0.5 | 7.14% (2.07)^2^ | 95.38% (0.05)^6^ | 3.08% (0.90)^3^ | 0.0153 (0.0128) |
|  |  | 1 | 12.44% (2.85)^6^ | 95.52% (0.06)^2^ | 5.40% (1.22)^3^ | 0.0485 (0.0175) |
|  |  | 2 | 30.59% (3.46)^6^ | 95.88% (0.08)^6^ | 13.26% (1.51)^4^ | 0.1613 (0.0212) |
|  |  | 3 | 58.78% (4.17)^5^ | 96.52% (0.12)^2^ | 26.02% (2.04)^1^ | 0.3420 (0.0269) |
|  |  | 4 | 84.10% (2.96)^5^ | 97.09% (0.09)^1^ | 37.37% (1.60)^1^ | 0.5034 (0.0197) |
|  |  | 5 | 97.25% (1.37)^5^ | 97.56% (0.08)^2^ | 45.07% (1.60)^6^ | 0.6049 (0.0161) |
|  | MMOM | 0.5 | 7.55% (2.23)^2^ | 95.29% (0.11)^6^ | 3.19% (0.94)^3^ | 0.0170 (0.0135) |
|  |  | 1 | 16.58% (3.04)^6^ | 95.52% (0.11)^2^ | 7.08% (1.27)^3^ | 0.0730 (0.0182) |
|  |  | 2 | 49.71% (3.74)^6^ | 95.93% (0.07)^6^ | 20.10% (1.44)^4^ | 0.2651 (0.0205 |
|  |  | 3 | 82.32% (3.20)^5^ | 96.52% (0.10)^2^ | 32.95% (1.26)^1^ | 0.4546 (0.0161) |
|  |  | 4 | 94.44% (2.72)^5^ | 97.22% (0.14)^1^ | 41.26% (1.54)^1^ | 0.5617 (0.0168) |
|  |  | 5 | 94.20% (3.58)^5^ | 97.56% (0.22)^2^ | 44.36% (2.34)^6^ | 0.5917 (0.0278) |
|  | COT | 0.5 | 27.69% (4.61)^1^ | 73.82% (2.55)^1^ | 15.68% (2.11)^1^ | 0.0114 (0.0332) |
|  |  | 1 | 38.06% (4.59)^1^ | 76.13% (2.32)^1^ | 21.94% (2.30)^1^ | 0.1089 (0.0349) |
|  |  | 2 | 77.09% (4.85)^1^ | 84.52% (2.45)^1^ | 46.98% (3.88)^1^ | 0.4871 (0.0452) |
|  |  | 3 | 96.31% (2.32)^1^ | 90.98% (1.48)^1^ | 65.45% (3.56)^1^ | 0.7305 (0.0317) |
|  |  | 4 | 99.57% (1.55)^1^ | 93.72% (1.58)^1^ | 73.92% (4.97)^1^ | 0.8157 (0.0403) |
|  |  | 5 | 99.55%^1^ (1.76) | 94.49%^1^ (1.60) | 76.44% (5.50)^1^ | 0.8356 (0.0434) |
|  | MMOT | 0.5 | 3.32% (1.41)1 | 96.14% (0.32)1 | 13.18% (5.61)^1^ | -0.0077 (0.0239) |
|  |  | 1 | 3.54% (1.47)^1^ | 96.16% (0.30)^1^ | 13.91% (5.54)^1^ | -0.0044 (0.0242) |
|  |  | 2 | 4.98% (1.53)^1^ | 96.42% (0.41)^1^ | 19.72% (6.00)^1^ | 0.0202 (0.0259) |
|  |  | 3 | 7.42% (1.89)^1^ | 96.83% (0.41)^1^ | 29.18% (7.08)^1^ | 0.0613 (0.0308) |
|  |  | 4 | 11.37% (2.35)^1^ | 97.37% (0.42)^1^ | 43.15% (6.67)^1^ | 0.1252 (0.0331) |
|  |  | 5 | 14.35%^1^ (2.61) | 97.80%^1^ (0.35) | 53.34% (6.58)^1^ | 0.1736 (0.0365) |
| **Type c** | sBIV | 0.5 | 1.76% (0.99)2 | 98.80% (0.01)1 | 2.94% (1.61)3 | 0.0070 (0.0124) |
|  |  | 1 | 3.31% (1.42)^1^ | 98.81% (0.02)^1^ | 5.40% (2.2)^1^ | 0.0262 (0.0175) |
|  |  | 2 | 7.18% (2.24)^1^ | 98.81% (0.02)^2^ | 10.99% (3.09)^6^ | 0.0720 (0.0261) |
|  |  | 3 | 14.07% (2.78)^4^ | 98.81% (0.02)^2^ | 19.50% (3.27)^4^ | 0.1490 (0.0304) |
|  |  | 4 | 24.11% (3.80)^4^ | 98.81% (0.02)^1^ | 29.26% (3.32)^4^ | 0.2506 (0.0364) |
|  |  | 5 | 33.29% (3.95)^1^ | 98.80% (0.02)^4^ | 36.38% (2.84)^4^ | 0.3346 (0.0331) |
|  | mBIV | 0.5 | 0.12% (0.28)^2^ | 99.97% (0.003)^1^ | 6.33% (15.31)^3^ | 0.0018 (0.0055) |
|  |  | 1 | 0.50% (0.51)^1^ | 99.97% (0.004)^1^ | 23.02% (21.60)^1^ | 0.0090 (0.0098) |
|  |  | 2 | 3.67% (1.53)^1^ | 99.97% (0.008)^2^ | 70.81% (10.24)^6^ | 0.0677 (0.0273) |
|  |  | 3 | 10.50% (2.26)^4^ | 99.97% (0.01)^2^ | 86.70% (4.07)^4^ | 0.1831 (0.0361) |
|  |  | 4 | 20.72% (3.83)^4^ | 99.96% (0.01)^1^ | 91.93% (2.96)^4^ | 0.3317 (0.0517) |
|  |  | 5 | 30.77% (3.89)^1^ | 99.96% (0.02)^4^ | 93.66% (2.34)^4^ | 0.4566 (0.0444) |
|  | SMOM | 0.5 | 5.68% (1.93)^2^ | 95.33% (0.04)^1^ | 2.46% (0.83)^3^ | 0.0061 (0.0119) |
|  |  | 1 | 8.94% (2.30)^1^ | 95.42% (0.06)^1^ | 3.87% (1.00)^1^ | 0.0265 (0.0143) |
|  |  | 2 | 14.70% (3.06)^1^ | 95.57% (0.07)^2^ | 6.42% (1.35)^6^ | 0.0629 (0.0191) |
|  |  | 3 | 24.46% (3.52)^4^ | 95.83% (0.10)^2^ | 10.80% (1.63)^4^ | 0.1253 (0.0226) |
|  |  | 4 | 35.65% (3.61)^4^ | 96.17% (0.12)^1^ | 16.08% (1.67)^4^ | 0.1993 (0.0230) |
|  |  | 5 | 44.90% (3.62)^1^ | 96.53% (0.14)^4^ | 21.09% (1.89)^4^ | 0.2667 (0.0250) |
|  | MMOM | 0.5 | 5.72% (1.78)^2^ | 95.20% (0.11)^1^ | 2.41% (0.76)^3^ | 0.0055 (0.0108) |
|  |  | 1 | 10.42% (2.73)^1^ | 95.34% (0.11)^1^ | 4.40% (1.13)^1^ | 0.0344 (0.0165) |
|  |  | 2 | 20.47% (3.46)^1^ | 95.58% (0.10)^2^ | 8.74% (1.45)^6^ | 0.0968 (0.0208) |
|  |  | 3 | 34.31% (3.32)^4^ | 95.84% (0.09)^2^ | 14.56% (1.36)^4^ | 0.1811 (0.0195) |
|  |  | 4 | 47.26% (4.16)^4^ | 96.09% (0.12)^1^ | 19.95% (1.58)^4^ | 0.2594 (0.0230) |
|  |  | 5 | 56.53% (3.62)^1^ | 96.43% (0.14)^4^ | 24.64% (1.54)^4^ | 0.3240 (0.0214) |
|  | COT | 0.5 | 26.09% (4.71)^1^ | 73.91% (2.69)^1^ | 14.93% (2.04)^7^ | -0.0002 (0.0318) |
|  |  | 1 | 28.08% (4.75)^1^ | 75.71% (2.54)^1^ | 16.93% (2.60)^7^ | 0.0300 (0.0393) |
|  |  | 2 | 38.01% (4.89)^1^ | 78.94% (2.16)^7^ | 24.14% (2.66)^1^ | 0.1370 (0.0390 |
|  |  | 3 | 49.26% (4.18)^1^ | 83.19% (2.33)^1^ | 34.24% (3.54)^1^ | 0.2748 (0.0421) |
|  |  | 4 | 60.08% (4.62)^1^ | 86.56% (2.20)^1^ | 44.32% (4.47)^1^ | 0.4066 (0.0497) |
|  |  | 5 | 67.98% (4.50)^1^ | 89.35% (2.000)^1^ | 53.24% (4.72)^1^ | 0.5140 (0.0472) |
|  | MMOT | 0.5 | 3.37% (1.43)^1^ | 96.26% (0.32)^1^ | 13.68% (5.77)^7^ | -0.0054 (0.0242) |
|  |  | 1 | 3.84% (1.57)^1^ | 96.30% (0.33)^1^ | 15.42% (6.13)^7^ | 0.0020 (0.0263) |
|  |  | 2 | 4.55% (1.65)^1^ | 96.36% (0.40)^7^ | 18.06% (6.52)^1^ | 0.0131 (0.0279) |
|  |  | 3 | 7.17% (2.02)^1^ | 96.69% (0.40)^1^ | 27.56% (7.43)^1^ | 0.0555 (0.0329) |
|  |  | 4 | 9.54% (2.15)^1^ | 96.93% (0.41)^1^ | 35.28% (6.81)^1^ | 0.0924 (0.0326) |
|  |  | 5 | 11.81% (2.22)^1^ | 97.26% (0.45)^1^ | 43.19% (6.81)^1^ | 0.1293 (0.0331) |
| **ALL** | sBIV | 0.5 | 2.17% (1.11)^1^ | 98.81% (0.03)^1^ | 5.18% (2.63)^2^ | 0.0136 (0.0157) |
|  |  | 1 | 3.41% (1.29)^3^ | 98.80% (0.02)^1^ | 7.76% (2.81)^2^ | 0.0303 (0.0177) |
|  |  | 2 | 10.85% (2.28)^3^ | 98.81% (0.02)^3^ | 21.36% (3.74)^1^ | 0.1267 (0.0285) |
|  |  | 3 | 26.55% (3.26)^1^ | 98.81% (0.03)^4^ | 40.13% (3.10)^3^ | 0.3031 (0.0333) |
|  |  | 4 | 44.57% (3.49)^1^ | 98.81% (0.03)^3^ | 52.69% (2.14)^3^ | 0.4686 (0.0291) |
|  |  | 5 | 58.62% (3.21)^1^ | 98.81% (0.02)^1^ | 59.56% (1.84)^3^ | 0.5785 (0.0244) |
|  | mBIV | 0.5 | 0.20% (0.26)^1^ | 99.97% (0.004)^1^ | 14.92% (18.66)^2^ | 0.0034 (0.0049) |
|  |  | 1 | 0.62% (0.55)^3^ | 99.97% (0.005)^1^ | 34.32% (22.39)^2^ | 0.0114 (0.0104) |
|  |  | 2 | 7.66% (1.96)^3^ | 99.96% (0.02)^3^ | 85.17% (6.06)^1^ | 0.1358 (0.0325) |
|  |  | 3 | 24.52% (3.04)^1^ | 99.94% (0.02)^4^ | 92.86% (2.56)^3^ | 0.3794 (0.0377) |
|  |  | 4 | 42.48% (3.31)^1^ | 99.94% (0.03)^3^ | 95.48% (2.00)^3^ | 0.5799 (0.0328) |
|  |  | 5 | 56.52% (3.31)^1^ | 99.94% (0.03)^1^ | 96.42% (1.44)^3^ | 0.7057 (0.0277) |
|  | SMOM | 0.5 | 6.73% (2.02)^1^ | 95.38% (0.06)^1^ | 4.21% (1.28)^2^ | 0.0164 (0.0160) |
|  |  | 1 | 9.97% (2.63)^3^ | 95.51% (0.07)^1^ | 6.19% (1.58)^2^ | 0.0422 (0.0204) |
|  |  | 2 | 26.19% (3.02)^3^ | 95.99% (0.10)^3^ | 16.41% (1.95)^1^ | 0.1720 (0.0239) |
|  |  | 3 | 47.66% (3.17)^1^ | 96.70% (0.11)^4^ | 30.38% (2.01)^3^ | 0.3476 (0.0240) |
|  |  | 4 | 66.78% (3.14)^1^ | 97.26% (0.09)^3^ | 42.11% (1.83)^3^ | 0.4986 (0.0221) |
|  |  | 5 | 78.09% (2.50)^1^ | 97.81% (0.11)^1^ | 51.71% (2.32)^3^ | 0.6082 (0.0220) |
|  | MMOM | 0.5 | 7.11% (1.71)^1^ | 95.31% (0.13)^1^ | 4.37% (1.09)^2^ | 0.0186 (0.0135) |
|  |  | 1 | 13.65% (2.39)^3^ | 95.56% (0.10)^1^ | 8.38% (1.41)^2^ | 0.070 (0.0183) |
|  |  | 2 | 39.83% (3.41)^3^ | 96.05% (0.10)^3^ | 23.25% (1.88)^1^ | 0.2664 (0.0241) |
|  |  | 3 | 62.77% (3.33)^1^ | 96.68% (0.14)^4^ | 36.32% (1.98)^3^ | 0.4391 (0.0235) |
|  |  | 4 | 70.08% (3.71)^1^ | 97.39% (0.14)^3^ | 44.47% (2.02)^3^ | 0.5272 (0.0248) |
|  |  | 5 | 60.46% (7.09)^1^ | 97.30% (0.24)^1^ | 40.15% (1.44)^3^ | 0.4634 (0.0277) |
|  | COT | 0.5 | 30.15% (4.40)^1^ | 74.39% (2.47)^1^ | 17.19% (2.27)^1^ | 0.0349 (0.0353) |
|  |  | 1 | 38.38% (4.78)^1^ | 77.40% (2.41)^1^ | 23.06% (2.57)^1^ | 0.1240 (0.0385) |
|  |  | 2 | 67.44% (4.14)^1^ | 87.14% (2.15)^1^ | 48.30% (4.24)^1^ | 0.4687 (0.0428) |
|  |  | 3 | 81.38% (3.82)^1^ | 93.44% (1.66)^1^ | 68.98% (5.54)^1^ | 0.6959 (0.0473) |
|  |  | 4 | 87.12% (2.79)^1^ | 96.32% (1.43)^1^ | 81.06% (6.10)^1^ | 0.8087 (0.0452) |
|  |  | 5 | 88.54% (2.71)^1^ | 98.01% (1.05)^1^ | 88.96% (5.21)^1^ | 0.8666 (0.0362) |
|  | MMOT | 0.5 | 4.05% (1.64)^1^ | 96.27% (0.38)^1^ | 16.08% (6.51)^1^ | 0.0046 (0.0280) |
|  |  | 1 | 3.98% (1.52)^1^ | 96.27% (0.38)^1^ | 15.87% (6.20)^1^ | 0.0036 (0.0263) |
|  |  | 2 | 6.74% (2.16)^1^ | 96.75% (0.33)^1^ | 26.55% (7.58)^1^ | 0.0504 (0.0340) |
|  |  | 3 | 12.18% (2.39)^1^ | 97.09% (0.37)^1^ | 42.29% (6.83)^1^ | 0.1310 (0.0354) |
|  |  | 4 | 17.56% (3.34)^1^ | 97.89% (0.40)^1^ | 59.18% (6.76)^1^ | 0.2169 (0.0443) |
|  |  | 5 | 15.73% (2.99)^1^ | 96.99% (0.58)^1^ | 48.00% (8.65)^1^ | 0.1758 (0.0461) |
| *Abbreviations: CTX, the co-trimoxazole prophylaxis trial; SD, standard deviation; sBIV, static WHO cut-off values for biologically implausible values detection method; mBIV, modified method for biologically implausible values detection; SMOM, single-model outlier measurement detection method; MMOM, multi-model outlier measurement detection method; COT, clustering-based outlier trajectory detection method;* *MMOT, multi-model outlier trajectory detection method.*  *^1^ p-value < 0.2% for the overall association and all possible combinations (mmom-mbiv, sbiv-mbiv, smom-mbiv, sBIV-mmom, smom-mmom, smom-sbiv) or < 0.2% for the comparison between cot and mmot*  *^2^ p-value < 0.2% for the overall association and for the combinations sbiv-mbiv, smom-mbiv only*  *^3^ p-value < 0.2% for the overall association and but not for combinations sbiv-mbiv*  *^4^p-value < 0.2% for the overall association and for combination sbiv-mmom only*  *^5^ p-value < 0.2% for the overall association and but not for all combinations*  *^6^ p-value < 0.2% for the overall association and but not for combinations mmom-mbiv and smom-mbiv*  *^7^p-value > 0.2% for the comparison between cot and mmot* | | | | | | |

| **Supplemental Table 4:** Sensitivity, specificity, precision and kappa per growth measure and dataset when combing outlier measurement detection methods (Method A and B). | | | | | | | |
| --- | --- | --- | --- | --- | --- | --- | --- |
| Dataset | Measure | Method A | Method B | Sensitivity | Specificity | Precision | Kappa |
| TARGet Kids! | zWFL | mBIV | MMOM | 9.5% | 96.4% | 32.1% | 8.4% |
| TARGet Kids! | zWFL | mBIV | MMOM | 14.0% | 97.1% | 45.8% | 15.5% |
| TARGet Kids! | zWFL | mBIV | MMOM | 33.1% | 98.8% | 82.5% | 42.2% |
| TARGet Kids! | zWFL | mBIV | MMOM | 46.0% | 99.1% | 90.4% | 56.6% |
| TARGet Kids! | zWFL | mBIV | MMOM | 58.3% | 99.4% | 94.5% | 68.5% |
| TARGet Kids! | zWFL | mBIV | MMOM | 78.8% | 99.4% | 95.9% | 84.4% |
| TARGet Kids! | zWFL | mBIV | SMOM | 8.1% | 96.2% | 27.3% | 6.0% |
| TARGet Kids! | zWFL | mBIV | SMOM | 12.5% | 97.2% | 43.7% | 13.7% |
| TARGet Kids! | zWFL | mBIV | SMOM | 28.2% | 98.8% | 80.1% | 36.8% |
| TARGet Kids! | zWFL | mBIV | SMOM | 43.4% | 99.3% | 91.1% | 54.4% |
| TARGet Kids! | zWFL | mBIV | SMOM | 60.8% | 99.6% | 96.6% | 71.3% |
| TARGet Kids! | zWFL | mBIV | SMOM | 73.7% | 99.7% | 97.8% | 81.7% |
| TARGet Kids! | zWFL | sBIV | MMOM | 9.5% | 96.4% | 31.9% | 8.4% |
| TARGet Kids! | zWFL | sBIV | MMOM | 14.0% | 97.0% | 45.5% | 15.5% |
| TARGet Kids! | zWFL | sBIV | MMOM | 33.3% | 98.8% | 82.6% | 42.5% |
| TARGet Kids! | zWFL | sBIV | MMOM | 46.0% | 99.1% | 90.0% | 56.4% |
| TARGet Kids! | zWFL | sBIV | MMOM | 59.3% | 99.3% | 94.0% | 69.1% |
| TARGet Kids! | zWFL | sBIV | MMOM | 80.7% | 99.4% | 96.0% | 85.7% |
| TARGet Kids! | zWFL | sBIV | SMOM | 8.1% | 96.2% | 27.3% | 6.0% |
| TARGet Kids! | zWFL | sBIV | SMOM | 12.5% | 97.2% | 43.7% | 13.7% |
| TARGet Kids! | zWFL | sBIV | SMOM | 28.2% | 98.8% | 80.1% | 36.8% |
| TARGet Kids! | zWFL | sBIV | SMOM | 43.4% | 99.2% | 90.7% | 54.3% |
| TARGet Kids! | zWFL | sBIV | SMOM | 60.8% | 99.6% | 96.3% | 71.2% |
| TARGet Kids! | zWFL | sBIV | SMOM | 73.9% | 99.7% | 97.5% | 81.7% |
| TARGet Kids! | zWFL | MMOM | SMOM | 11.4% | 94.8% | 28.1% | 8.3% |
| TARGet Kids! | zWFL | MMOM | SMOM | 17.0% | 96.0% | 42.8% | 17.3% |
| TARGet Kids! | zWFL | MMOM | SMOM | 39.6% | 98.3% | 80.3% | 47.9% |
| TARGet Kids! | zWFL | MMOM | SMOM | 55.1% | 98.9% | 89.7% | 64.1% |
| TARGet Kids! | zWFL | MMOM | SMOM | 71.2% | 99.4% | 95.5% | 78.8% |
| TARGet Kids! | zWFL | MMOM | SMOM | 79.0% | 99.5% | 96.6% | 84.9% |
| CTX | zWA | mBIV | MMOM | 5.0% | 95.4% | 16.0% | 0.5% |
| CTX | zWA | mBIV | MMOM | 12.1% | 96.4% | 37.4% | 11.8% |
| CTX | zWA | mBIV | MMOM | 29.2% | 98.1% | 73.1% | 36.3% |
| CTX | zWA | mBIV | MMOM | 47.3% | 99.0% | 89.3% | 57.4% |
| CTX | zWA | mBIV | MMOM | 64.6% | 99.3% | 94.1% | 73.4% |
| CTX | zWA | mBIV | MMOM | 71.8% | 99.7% | 97.6% | 80.2% |
| CTX | zWA | mBIV | SMOM | 4.7% | 95.3% | 15.1% | 0.1% |
| CTX | zWA | mBIV | SMOM | 9.7% | 96.4% | 31.9% | 8.5% |
| CTX | zWA | mBIV | SMOM | 19.2% | 97.5% | 57.7% | 23.1% |
| CTX | zWA | mBIV | SMOM | 33.0% | 98.7% | 81.3% | 41.9% |
| CTX | zWA | mBIV | SMOM | 54.9% | 99.4% | 93.9% | 65.4% |
| CTX | zWA | mBIV | SMOM | 69.5% | 99.6% | 97.2% | 78.4% |
| CTX | zWA | sBIV | MMOM | 5.1% | 95.2% | 15.7% | 0.4% |
| CTX | zWA | sBIV | MMOM | 12.2% | 96.3% | 36.6% | 11.7% |
| CTX | zWA | sBIV | MMOM | 29.8% | 97.7% | 69.7% | 36.0% |
| CTX | zWA | sBIV | MMOM | 48.3% | 98.2% | 82.9% | 56.2% |
| CTX | zWA | sBIV | MMOM | 66.1% | 98.3% | 87.2% | 71.5% |
| CTX | zWA | sBIV | MMOM | 73.3% | 98.8% | 91.6% | 78.6% |
| CTX | zWA | sBIV | SMOM | 4.7% | 95.3% | 15.1% | 0.1% |
| CTX | zWA | sBIV | SMOM | 9.7% | 96.3% | 31.8% | 8.4% |
| CTX | zWA | sBIV | SMOM | 19.6% | 97.3% | 56.3% | 23.1% |
| CTX | zWA | sBIV | SMOM | 33.7% | 98.3% | 77.7% | 41.7% |
| CTX | zWA | sBIV | SMOM | 55.8% | 98.6% | 87.7% | 64.0% |
| CTX | zWA | sBIV | SMOM | 70.5% | 98.8% | 91.5% | 76.6% |
| CTX | zWA | MMOM | SMOM | 6.1% | 94.4% | 16.1% | 0.7% |
| CTX | zWA | MMOM | SMOM | 13.5% | 95.7% | 35.7% | 12.4% |
| CTX | zWA | MMOM | SMOM | 31.8% | 97.3% | 67.7% | 37.2% |
| CTX | zWA | MMOM | SMOM | 46.4% | 98.6% | 85.4% | 55.4% |
| CTX | zWA | MMOM | SMOM | 61.2% | 99.2% | 93.1% | 70.3% |
| CTX | zWA | MMOM | SMOM | 67.3% | 99.9% | 98.8% | 77.3% |
| CTX | zMUAC | mBIV | MMOM | 9.0% | 95.8% | 27.5% | 6.6% |
| CTX | zMUAC | mBIV | MMOM | 13.3% | 96.4% | 39.7% | 13.4% |
| CTX | zMUAC | mBIV | MMOM | 26.5% | 98.4% | 74.9% | 34.0% |
| CTX | zMUAC | mBIV | MMOM | 46.5% | 99.2% | 91.0% | 57.2% |
| CTX | zMUAC | mBIV | MMOM | 64.1% | 99.8% | 98.3% | 74.6% |
| CTX | zMUAC | mBIV | MMOM | 71.2% | 100.0% | 99.7% | 80.7% |
| CTX | zMUAC | mBIV | SMOM | 9.0% | 95.5% | 26.1% | 6.2% |
| CTX | zMUAC | mBIV | SMOM | 12.3% | 96.3% | 37.2% | 11.9% |
| CTX | zMUAC | mBIV | SMOM | 22.9% | 97.9% | 66.1% | 28.5% |
| CTX | zMUAC | mBIV | SMOM | 37.8% | 99.1% | 87.8% | 48.1% |
| CTX | zMUAC | mBIV | SMOM | 51.9% | 99.8% | 97.6% | 64.0% |
| CTX | zMUAC | mBIV | SMOM | 67.9% | 99.9% | 99.5% | 78.0% |
| CTX | zMUAC | sBIV | MMOM | 9.0% | 95.8% | 27.5% | 6.6% |
| CTX | zMUAC | sBIV | MMOM | 13.3% | 96.4% | 39.7% | 13.4% |
| CTX | zMUAC | sBIV | MMOM | 26.6% | 98.4% | 74.4% | 34.0% |
| CTX | zMUAC | sBIV | MMOM | 47.1% | 99.1% | 89.9% | 57.4% |
| CTX | zMUAC | sBIV | MMOM | 64.7% | 99.7% | 97.8% | 74.9% |
| CTX | zMUAC | sBIV | MMOM | 72.5% | 99.9% | 99.0% | 81.3% |
| CTX | zMUAC | sBIV | SMOM | 9.0% | 95.5% | 26.1% | 6.2% |
| CTX | zMUAC | sBIV | SMOM | 12.3% | 96.3% | 37.2% | 11.9% |
| CTX | zMUAC | sBIV | SMOM | 22.9% | 97.9% | 66.1% | 28.5% |
| CTX | zMUAC | sBIV | SMOM | 37.9% | 99.0% | 87.4% | 48.1% |
| CTX | zMUAC | sBIV | SMOM | 52.1% | 99.7% | 97.1% | 64.0% |
| CTX | zMUAC | sBIV | SMOM | 68.2% | 99.9% | 98.9% | 78.1% |
| CTX | zMUAC | MMOM | SMOM | 12.7% | 94.0% | 27.3% | 8.7% |
| CTX | zMUAC | MMOM | SMOM | 16.7% | 95.2% | 38.2% | 15.5% |
| CTX | zMUAC | MMOM | SMOM | 34.2% | 97.2% | 68.1% | 39.4% |
| CTX | zMUAC | MMOM | SMOM | 56.0% | 98.6% | 87.3% | 64.0% |
| CTX | zMUAC | MMOM | SMOM | 68.8% | 99.7% | 97.4% | 77.9% |
| CTX | zMUAC | MMOM | SMOM | 75.0% | 99.9% | 99.5% | 83.4% |
| Abbreviations: CTX, the co-trimoxazole prophylaxis trial; sBIV, static WHO cut-off values for biologically implausible values detection method; mBIV, modified method for biologically implausible values detection; SMOM, single-model outlier measurement detection method; MMOM, multi-model outlier measurement detection method; zWFL ; weight-for-length z-scores ; zWA, weight-for-age z-scores; zMUAC, mid-upper arm circumference-for-age z-scores; | | | | | | | |

| **Supplemental Table 5**: Model fitting parameters for the population average trajectory for the original dataset and for the dataset with outliers of 6 different intensities | | | | | |
| --- | --- | --- | --- | --- | --- |
| **Data** | **Measure** | **Type Error** | **SD** | **Original dataset RMSE** | **Outlier dataset RMSE** |
| **TARGet Kids!** | zWA | ALL | 1 | 1.057516 | 1.175453 |
| **TARGet Kids!** | zWA | ALL | 3 |  | 1.632279 |
| **TARGet Kids!** | zWA | ALL | 5 |  | 2.330279 |
| **CTX** | zWA | ALL | 1 | 0.9575387 | 1.015789 |
| **CTX** | zWA | ALL | 3 |  | 1.376043 |
| **CTX** | zWA | ALL | 5 |  | 1.925456 |
| Abbreviations: CTX, the co-trimoxazole prophylaxis trial; zWA, weight-for-age z-scores; SD, standard deviation; RMSE, Root Mean Square Error; ALL, all types of errors | | | | | |

**Supplemental Table 6**: Summary of sensitivity results with 4 outliers per trajectory applied on the TARGet Kids! dataset.

| **Outlier type** | **Detection method** | **SD error** | **Sensitivity** | **Specificity** | **Precision** | **Kappa** |
| --- | --- | --- | --- | --- | --- | --- |
| Type a | sBIV | 0.5 | 0.79% | 99.69% | 5.44% | 0.00825 |
|  |  | 1 | 0.76% | 99.69% | 5.13% | 0.00772 |
|  |  | 2 | 1.67% | 99.69% | 10.25% | 0.02293 |
|  |  | 3 | 6.04% | 99.69% | 29.75% | 0.09364 |
|  |  | 4 | 21.59% | 99.69% | 60.77% | 0.30959 |
|  |  | 5 | 49.61% | 99.69% | 78.35% | 0.59943 |
|  | mBIV | 0.5 | 0.72% | 99.72% | 5.54% | 0.00771 |
|  |  | 1 | 0.69% | 99.72% | 5.19% | 0.00714 |
|  |  | 2 | 1.63% | 99.72% | 10.95% | 0.02300 |
|  |  | 3 | 5.96% | 99.72% | 31.60% | 0.09385 |
|  |  | 4 | 21.49% | 99.72% | 63.12% | 0.31182 |
|  |  | 5 | 49.33% | 99.73% | 80.29% | 0.60315 |
|  | SMOM | 0.5 | 6.29% | 95.60% | 3.14% | 0.01265 |
|  |  | 1 | 11.78% | 95.69% | 5.80% | 0.04962 |
|  |  | 2 | 35.01% | 96.11% | 16.96% | 0.20456 |
|  |  | 3 | 63.23% | 96.68% | 30.34% | 0.39136 |
|  |  | 4 | 85.92% | 97.31% | 42.28% | 0.55305 |
|  |  | 5 | 95.41% | 97.90% | 50.70% | 0.65176 |
|  | MMOM | 0.5 | 7.40% | 95.82% | 3.86% | 0.02222 |
|  |  | 1 | 14.12% | 95.93% | 7.26% | 0.06864 |
|  |  | 2 | 43.21% | 96.40% | 21.43% | 0.26443 |
|  |  | 3 | 74.94% | 96.99% | 36.31% | 0.47305 |
|  |  | 4 | 92.72% | 97.70% | 48.09% | 0.62184 |
|  |  | 5 | 98.28% | 98.24% | 55.85% | 0.70338 |
|  | COT | 0.5 | 24.81% | 75.57% | 15.24% | 0.00319 |
|  |  | 1 | 27.14% | 75.84% | 16.64% | 0.02392 |
|  |  | 2 | 42.47% | 77.99% | 25.46% | 0.15996 |
|  |  | 3 | 63.68% | 82.53% | 39.48% | 0.36799 |
|  |  | 4 | 82.19% | 87.84% | 55.09% | 0.58137 |
|  |  | 5 | 92.83% | 90.48% | 64.16% | 0.70216 |
|  | MMOT | 0.5 | 5.90% | 94.67% | 16.31% | 0.00770 |
|  |  | 1 | 6.22% | 94.72% | 17.20% | 0.01271 |
|  |  | 2 | 10.90% | 96.02% | 32.69% | 0.09524 |
|  |  | 3 | 19.54% | 97.70% | 60.07% | 0.23820 |
|  |  | 4 | 28.19% | 98.87% | 81.44% | 0.36921 |
|  |  | 5 | 34.58% | 99.54% | 92.98% | 0.45886 |
| Type b | sBIV | 0.5 | 0.52% | 99.68% | 3.43% | 0.00344 |
|  |  | 1 | 0.82% | 99.68% | 5.22% | 0.00849 |
|  |  | 2 | 2.86% | 99.68% | 16.12% | 0.04251 |
|  |  | 3 | 11.68% | 99.68% | 44.04% | 0.17655 |
|  |  | 4 | 42.71% | 99.68% | 75.00% | 0.53542 |
|  |  | 5 | 99.67% | 99.68% | 87.62% | 0.93092 |
|  | mBIV | 0.5 | 0.41% | 99.71% | 3.00% | 0.00207 |
|  |  | 1 | 0.65% | 99.71% | 4.65% | 0.00622 |
|  |  | 2 | 2.65% | 99.72% | 16.40% | 0.03999 |
|  |  | 3 | 11.50% | 99.71% | 46.15% | 0.17640 |
|  |  | 4 | 42.09% | 99.71% | 76.75% | 0.53488 |
|  |  | 5 | 98.65% | 99.72% | 88.79% | 0.93296 |
|  | SMOM | 0.5 | 10.17% | 95.71% | 5.10% | 0.03948 |
|  |  | 1 | 21.68% | 95.96% | 10.90% | 0.11882 |
|  |  | 2 | 65.69% | 96.52% | 29.82% | 0.39156 |
|  |  | 3 | 89.73% | 97.15% | 41.71% | 0.55572 |
|  |  | 4 | 98.90% | 97.80% | 50.72% | 0.66025 |
|  |  | 5 | 100.00% | 98.34% | 57.95% | 0.72576 |
|  | MMOM | 0.5 | 11.75% | 95.95% | 6.19% | 0.05344 |
|  |  | 1 | 24.91% | 96.14% | 12.84% | 0.14413 |
|  |  | 2 | 71.33% | 96.77% | 33.23% | 0.43618 |
|  |  | 3 | 92.61% | 97.45% | 45.20% | 0.59511 |
|  |  | 4 | 98.33% | 98.08% | 53.99% | 0.68770 |
|  |  | 5 | 99.86% | 98.52% | 60.77% | 0.74818 |
|  | COT | 0.5 | 26.15% | 75.83% | 16.10% | 0.01610 |
|  |  | 1 | 32.02% | 76.83% | 19.68% | 0.07035 |
|  |  | 2 | 48.53% | 80.31% | 30.64% | 0.23230 |
|  |  | 3 | 72.75% | 84.28% | 45.57% | 0.45675 |
|  |  | 4 | 89.83% | 89.13% | 59.89% | 0.65391 |
|  |  | 5 | 96.12% | 91.95% | 68.40% | 0.75364 |
|  | MMOT | 0.5 | 6.22% | 94.64% | 17.04% | 0.01168 |
|  |  | 1 | 8.61% | 95.30% | 24.48% | 0.05325 |
|  |  | 2 | 15.53% | 97.17% | 49.04% | 0.17627 |
|  |  | 3 | 24.17% | 98.53% | 74.29% | 0.31356 |
|  |  | 4 | 32.42% | 99.31% | 89.25% | 0.42898 |
|  |  | 5 | 35.58% | 99.72% | 95.79% | 0.47494 |
| Type c | sBIV | 0.5 | 0.76% | 99.68% | 4.97% | 0.00749 |
|  |  | 1 | 1.28% | 99.68% | 7.96% | 0.01621 |
|  |  | 2 | 6.15% | 99.68% | 29.18% | 0.09467 |
|  |  | 3 | 18.88% | 99.68% | 56.71% | 0.27433 |
|  |  | 4 | 35.41% | 99.68% | 71.47% | 0.46435 |
|  |  | 5 | 51.85% | 99.68% | 78.62% | 0.61693 |
|  | mBIV | 0.5 | 0.68% | 99.71% | 4.95% | 0.00686 |
|  |  | 1 | 1.15% | 99.72% | 7.97% | 0.01485 |
|  |  | 2 | 5.95% | 99.72% | 30.81% | 0.09330 |
|  |  | 3 | 18.60% | 99.72% | 59.07% | 0.27422 |
|  |  | 4 | 35.08% | 99.71% | 73.29% | 0.46547 |
|  |  | 5 | 51.38% | 99.71% | 80.17% | 0.61846 |
|  | SMOM | 0.5 | 10.03% | 95.70% | 5.03% | 0.03853 |
|  |  | 1 | 21.75% | 95.94% | 10.88% | 0.11878 |
|  |  | 2 | 50.02% | 96.51% | 24.53% | 0.30835 |
|  |  | 3 | 69.63% | 97.10% | 35.54% | 0.45399 |
|  |  | 4 | 81.69% | 97.75% | 45.29% | 0.56988 |
|  |  | 5 | 88.85% | 98.24% | 53.62% | 0.65843 |
|  | MMOM | 0.5 | 11.61% | 95.92% | 6.06% | 0.05193 |
|  |  | 1 | 24.26% | 96.15% | 12.58% | 0.14037 |
|  |  | 2 | 52.83% | 96.69% | 26.58% | 0.33380 |
|  |  | 3 | 73.96% | 97.25% | 38.22% | 0.48845 |
|  |  | 4 | 86.30% | 97.83% | 47.65% | 0.60197 |
|  |  | 5 | 91.19% | 98.32% | 55.39% | 0.67954 |
|  | COT | 0.5 | 26.81% | 76.10% | 16.56% | 0.02324 |
|  |  | 1 | 31.34% | 77.30% | 19.72% | 0.06973 |
|  |  | 2 | 41.56% | 81.08% | 28.23% | 0.18954 |
|  |  | 3 | 53.56% | 85.08% | 39.48% | 0.33627 |
|  |  | 4 | 65.47% | 88.06% | 49.72% | 0.47239 |
|  |  | 5 | 74.22% | 90.95% | 59.94% | 0.59189 |
|  | MMOT | 0.5 | 7.31% | 95.00% | 20.63% | 0.03150 |
|  |  | 1 | 9.17% | 95.73% | 27.48% | 0.06741 |
|  |  | 2 | 16.03% | 97.40% | 52.18% | 0.18751 |
|  |  | 3 | 21.63% | 98.65% | 74.34% | 0.28502 |
|  |  | 4 | 26.27% | 99.53% | 91.34% | 0.36313 |
|  |  | 5 | 26.75% | 99.96% | 99.23% | 0.38019 |
| ALL | sBIV | 0.5 | 0.61% | 99.68% | 5.56% | 0.00512 |
|  |  | 1 | 1.24% | 99.69% | 10.92% | 0.01629 |
|  |  | 2 | 4.24% | 99.69% | 29.03% | 0.06682 |
|  |  | 3 | 13.62% | 99.69% | 57.50% | 0.21027 |
|  |  | 4 | 34.62% | 99.69% | 77.23% | 0.46687 |
|  |  | 5 | 67.71% | 99.69% | 87.23% | 0.75504 |
|  | mBIV | 0.5 | 0.51% | 99.71% | 5.21% | 0.00390 |
|  |  | 1 | 1.12% | 99.72% | 10.91% | 0.01487 |
|  |  | 2 | 3.93% | 99.73% | 30.05% | 0.06302 |
|  |  | 3 | 13.01% | 99.72% | 59.11% | 0.20381 |
|  |  | 4 | 33.69% | 99.72% | 78.83% | 0.46105 |
|  |  | 5 | 65.89% | 99.72% | 88.21% | 0.74682 |
|  | SMOM | 0.5 | 9.19% | 95.72% | 6.39% | 0.04031 |
|  |  | 1 | 19.04% | 95.99% | 13.12% | 0.12319 |
|  |  | 2 | 48.96% | 96.68% | 32.03% | 0.36301 |
|  |  | 3 | 73.20% | 97.43% | 47.88% | 0.56174 |
|  |  | 4 | 87.78% | 98.11% | 59.29% | 0.69632 |
|  |  | 5 | 93.84% | 98.68% | 69.24% | 0.78889 |
|  | MMOM | 0.5 | 10.63% | 95.94% | 7.69% | 0.05525 |
|  |  | 1 | 21.55% | 96.21% | 15.31% | 0.14812 |
|  |  | 2 | 53.78% | 96.91% | 35.80% | 0.40740 |
|  |  | 3 | 78.37% | 97.68% | 52.14% | 0.61108 |
|  |  | 4 | 90.32% | 98.33% | 62.99% | 0.73212 |
|  |  | 5 | 95.30% | 98.86% | 72.73% | 0.81811 |
|  | COT | 0.5 | 28.36% | 75.87% | 17.18% | 0.03312 |
|  |  | 1 | 35.47% | 77.23% | 21.67% | 0.10058 |
|  |  | 2 | 51.93% | 81.87% | 33.82% | 0.27641 |
|  |  | 3 | 67.37% | 87.40% | 49.26% | 0.47451 |
|  |  | 4 | 79.88% | 91.35% | 62.61% | 0.63778 |
|  |  | 5 | 86.64% | 94.19% | 73.19% | 0.74967 |
|  | MMOT | 0.5 | 6.10% | 94.67% | 16.81% | 0.01041 |
|  |  | 1 | 9.32% | 95.54% | 26.93% | 0.06640 |
|  |  | 2 | 18.27% | 97.49% | 56.08% | 0.21755 |
|  |  | 3 | 27.22% | 98.97% | 82.33% | 0.35994 |
|  |  | 4 | 31.78% | 99.81% | 96.72% | 0.43520 |
|  |  | 5 | 32.98% | 100.00% | 99.94% | 0.45363 |
| *Abbreviations: SD, standard deviation; sBIV, static WHO cut-off values for biologically implausible values detection method; mBIV, modified method for biologically implausible values detection; SMOM, single-model outlier measurement detection method; MMOM, multi-model outlier measurement detection method; COT, clustering-based outlier trajectory detection method;* *MMOT, multi-model outlier trajectory detection method.* | | | | | | |

**References**

1. Kuhn M. Building Predictive Models in R Using the caret Package. *2008* 2008;28(5):26.

2. Altman DG, Bland JM. Diagnostic tests. 1: Sensitivity and specificity. *BMJ* 1994;308(6943):1552.

3. Altman DG, Bland JM. Diagnostic tests 2: Predictive values. *BMJ* 1994;309(6947):102.
